# Gene-rich germline-restricted chromosomes in black-winged fungus gnats evolved through hybridization

**DOI:** 10.1371/journal.pbio.3001559

**Published:** 2022-02-25

**Authors:** Christina N. Hodson, Kamil S. Jaron, Susan Gerbi, Laura Ross

**Affiliations:** 1 Institute of Evolutionary Biology, University of Edinburgh, Edinburgh, United Kingdom; 2 Division of Biology and Medicine, Brown University, Providence, Rhode Island, United States of America; University of Cambridge, UNITED KINGDOM

## Abstract

Germline-restricted DNA has evolved in diverse animal taxa and is found in several vertebrate clades, nematodes, and flies. In these lineages, either portions of chromosomes or entire chromosomes are eliminated from somatic cells early in development, restricting portions of the genome to the germline. Little is known about why germline-restricted DNA has evolved, especially in flies, in which 3 diverse families, Chironomidae, Cecidomyiidae, and Sciaridae, carry germline-restricted chromosomes (GRCs). We conducted a genomic analysis of GRCs in the fungus gnat *Bradysia* (*Sciara*) *coprophila* (Diptera: Sciaridae), which has 2 large germline-restricted “L” chromosomes. We sequenced and assembled the genome of *B*. *coprophila* and used differences in sequence coverage and k-mer frequency between somatic and germline tissues to identify GRC sequence and compare it to the other chromosomes in the genome. We found that the GRCs in *B*. *coprophila* are large, gene rich, and have many genes with divergent homologs on other chromosomes in the genome. We also found that 2 divergent GRCs exist in the population we sequenced. GRC genes are more similar in sequence to genes from another Dipteran family (Cecidomyiidae) than to homologous genes from Sciaridae. This unexpected finding suggests that these chromosomes likely arose in Sciaridae through hybridization with a related lineage. These results provide a foundation from which to answer many questions about the evolution of GRCs in Sciaridae, such as how this hybridization event resulted in GRCs and what features on these chromosomes cause them to be restricted to the germline.

## Abstract 2

Germ-line restricted chromosomes are eliminated from all somatic tissues while being retained in the germline. This study of the evolutionary origin of such chromosomes in fungus gnats reveals that they are most similar to a fly of a different family, suggesting an ancient allopolyploidization origin for these peculiar chromosomes.

## Introduction

An underlying tenet of heredity is that all cells within an organism have the same genomic sequence. However, there are a surprising number of exceptions to this rule. For instance, Boveri [1] noted in *Ascaris* nematodes that fragments of chromosomes were eliminated from somatic cells early in development, showing that, in some cases, germline/soma differentiation involves changes in the genomic composition of cells as well as regulatory changes. In addition to the loss of chromosomal fragments (referred to as “chromatin diminution”), another type of germline specialization involves the elimination of whole chromosomes from somatic cells, a phenomenon identified in the Dipteran gnat *Bradysia* (*Sciara*) *coprophila* nearly a century ago [2]. Both chromatin diminution and chromosome elimination are examples of programmed DNA elimination, which occurs in a developmentally regulated manner across a broad evolutionary range from ciliates to mammals, including thousands of species from 9 major taxonomic groups [3]. Programmed DNA elimination is not a rare phenomenon, yet it remains poorly understood. Recently, however, genomic studies in several species are beginning to address questions regarding the function and evolution of programmed DNA elimination.

Many examples of programmed DNA elimination involve regulated DNA elimination from somatic cells so that portions of the genome are restricted to the germline [3]. Germline-restricted DNA, involving either portions of chromosomes (chromatin diminution) or entire chromosomes (chromosome elimination), has evolved repeatedly and is found in lampreys and hagfish (early branching vertebrates), songbirds, nematodes, ciliates, copepods, and flies [1,3–9]. Recent genomic work on lampreys and nematodes (with chromatin diminution) and songbirds (with chromosome elimination) have found that the germline-restricted portions of the genome often carry protein coding genes involved in germline tissue maturation and function [10–13]. Therefore, a leading hypothesis is that germline-restricted DNA may help resolve conflict between the germline and somatic cells, such that germline-restricted DNA evolves to either restrict portions of the genome to the germline that are harmful in somatic cells or as a means of germline specialization [3,14]. Alternatively, the observation that repetitive DNA and transposable elements are often among germline-restricted portions of the genome in species with chromatin diminution, and that germline-restricted chromosomes (GRCs) sometimes show variation in number and often show sex-biased transmission patterns, suggests that germline-restricted DNA may evolve from selfish genetic entities [9,15–17]. Understanding more about the evolution of germline-restricted DNA in different lineages can help us understand whether germline-restricted DNA evolves through selfish or adaptive means and whether different origins of germline-restricted DNA show similar patterns of evolution. Genomic work focused on understanding germline-restricted DNA has taken place in nematodes, lampreys, ciliates, and zebra finches. Importantly, only one of these lineages, zebra finches, exhibits chromosome elimination, so we still know little about how these 2 types of germline-restricted DNA compare to each other.

In species with chromosome elimination, entire chromosomes are exclusively found in the germline: GRCs. One hypothesis for the evolution of GRCs is that they originate from B chromosomes [18], which are accessory nonessential chromosomes that are widespread in eukaryotes [19]. GRCs are similar to B chromosomes in that they are chromosomes in addition to the core genome (i.e., the chromosomes which are found in the somatic cells as well as the germline cells), with greater variation in presence/number of chromosomes than the core chromosome set. However, while B chromosomes are nonessential, recent genomic work in songbirds suggests that GRCs likely play an important, and perhaps fundamental, role in zebra finches [13],and are evolutionarily conserved across songbirds [20]. Furthermore, there is no clear evidence that GRCs spread through drive and therefore, unlike B chromosomes, they most likely persist due to their functional importance, rather than as reproductive parasites. So while it is possible that GRCs originated from B chromosomes and were subsequently “domesticated,” alternative explanations for their origin cannot be excluded and should be explored, especially as the origins of the GRCs have so far only focused on their single origin among birds. Here, we focus on a different origin of GRCs: their evolution and origin in flies (Diptera).

GRCs are found in 3 Dipteran families: the “K” chromosomes of nonbiting midges (Chironomidae), the “E” chromosomes of gall gnats (Cecidomyiidae), and the “L” chromosomes of black winged fungus gnats (Sciaridae) [4,21,22]. Each instance appears to have an independent origin, as GRCs vary in number, size, and transmission patterns in each lineage, and the 3 families are not sister clades [23–25]. The origin and evolution of GRCs in Sciaridae and Cecidomyiidae are particularly intriguing, as these families are relatively closely related, both belonging to the infraorder Bibionomorpha (although they are not sister clades [24]). Understanding how GRCs arose in these 2 lineages and what factors led to their evolution can provide a foundation from which we can answer many questions. For instance, we can start to unravel why GRCs arose in some Dipteran families but not others and compare the gene content and expression of GRC genes in 2 relatively closely related families.

**Fig 1:**
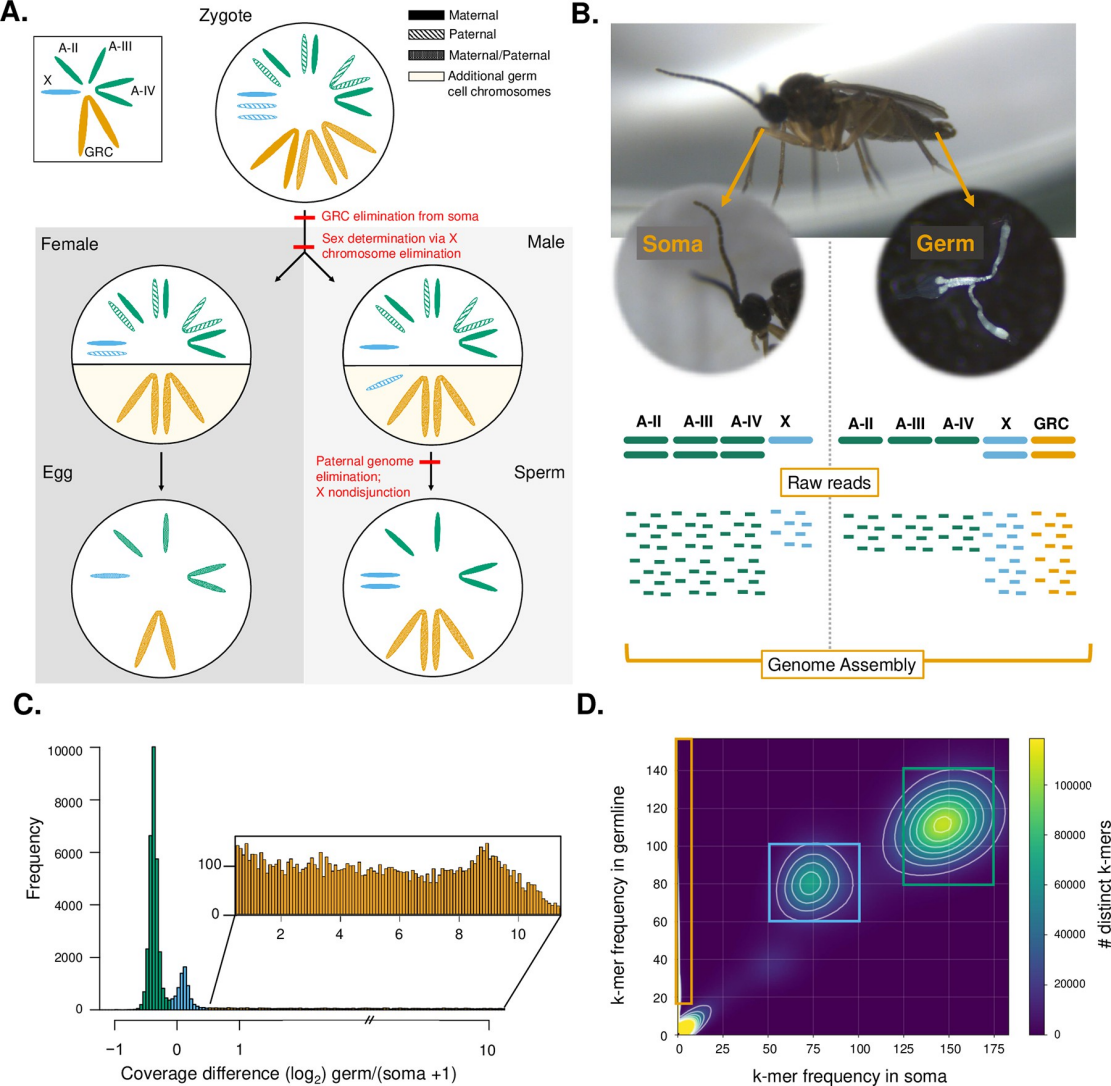
GRC transmission and sequencing and identification of GRCs through comparison of germline and soma coverage. **(A)** *B*. *coprophila* has 3 autosomes (A, green), an XO sex determination system (X chromosome shown in blue), and GRC (orange). Paternal origin chromosomes = dashed, maternal origin chromosomes = solid, either maternal or paternal origin chromosomes = dotted. Chromosomes below the solid line in males and females are additional chromosomes present in germline cells but eliminated from somatic cells. *B*. *coprophila* GRCs are eliminated from somatic cells in early embryogenesis. Some X chromosomes (always paternally inherited) are also eliminated from somatic cells early in development to determine sex. Males undergo paternal genome elimination such that (apart from the GRCs) only maternally inherited chromosomes are transmitted through the sperm (including 2 copies of the maternally derived X chromosome due to a nondisjunction event in meiosis). Chromosome sizes and shapes approximated from [21]. **(B)** Schematic of sequencing approach for identifying GRC sequences in *B*. *coprophila*. We isolated and sequenced somatic (head) and germline (testes with sperm) tissue, which differ in the number of autosomes (A-II, A-III, and A-IV), X chromosomes, and GRCs. We used the differences in chromosome constitution to isolate regions belonging to each chromosome type in a genome assembly made from short-read sequences from both tissue types. **(C)** Histogram of per scaffold log_2_ coverage differences between germline and somatic tissues. Green regions were assigned as autosomal, blue assigned as the X chromosome, and orange assigned as the GRCs (inset). Note that 1 was added to the number of somatic reads to ensure noninfinite log_2_ coverage differences for GRC scaffolds. **(D)** Comparison of k-mer (27-mer) frequency differences between reads in the germline and soma libraries. K-mers were mapped to the genome assembly and scaffolds assigned based on which type of k-mers (GRC, X chromosome, or autosomal) mapped to the scaffold and what proportion of the scaffold had k-mers mapping to it (**S2 Fig**). Boxes show coverage of k-mers assigned as autosomal (green), X chromosome (blue), and GRC (orange). Location of data used to generate Fig 1C and 1D is specified in **S1 Table**. GRC, germline-restricted chromosome.

Although both Sciaridae and Cecidomyiidae carry GRCs, the characteristics of these chromosomes differ between the 2 families, with Sciaridae carrying few (up to 4) large GRCs, and Cecidomyiidae carrying many (between 16 and 67) small GRCs (reviewed in [23,25,26]). Therefore, hypotheses for how GRCs arose differ between the 2 lineages. In Cecidomyiidae, the GRCs show some similarities in appearance to the core genome, and so it was originally proposed that they evolved through whole genome duplications followed by restriction of the duplicated chromosomes to the germline [27,28], although this idea remains controversial and lacks empirical support [29]. In Sciaridae, however, it has been hypothesized that the GRCs evolved from the X chromosome in a series of conflicts between different parts of the genome [30]. This hypothesis suggests that the evolution of GRCs is closely intertwined to the unusual genetic system found in this lineage. Sciarids display a non-mendelian chromosome inheritance system known as paternal genome elimination [21,31] and have an XO sex chromosome system. In species with paternal genome elimination meiosis in males is unconventional, such that males only transmit chromosomes that they inherit from their mother to their offspring, while paternal chromosomes are eliminated during male meiosis. In addition, in male meiosis in *B*. *coprophila*, all GRCs present (normally 2) are transmitted to offspring regardless of parental origin [32], and there is an unusual X chromosome nondisjunction event such that 2 copies of the X chromosome are transmitted through sperm. This results in males transmitting typically 2 GRCs, 2 X chromosomes, and a haploid set of autosomes through sperm (**Fig 1**). Furthermore, in Sciaridae, sex is determined by the number of X chromosomes eliminated from somatic cells in embryogenesis [2,33]. Sex chromosome elimination occurs early in development, when the X chromosome(s) that will be eliminated are left on the metaphase plate and not incorporated into daughter nuclei. GRCs are eliminated from somatic cells in a similar way, with the exception that GRC elimination occurs slightly earlier in development than X chromosome elimination [2] (**Fig 1**; see **S1 Text** for additional information).

The hypothesis for the evolution of this unusual genetic system suggests that paternal genome elimination and X chromosome elimination as a means of sex determination evolved at the base of the Sciaridae [30]. Following this, GRCs evolved from the paternally derived X chromosome in males as a means to escape elimination through paternal genome elimination. This was followed by restriction of this chromosome to the germline as X chromosome polyploidy in the somatic cells might be detrimental. Although there has been no attempt to validate this idea in Sciaridae, it contains some testable predictions. For instance, following this hypothesis [30], we would expect that GRCs, if they were derived from the X chromosome, would exhibit homology to this chromosome and that the GRCs would have evolved recently, in the common ancestor of Sciaridae, as GRCs are found in diverse Sciarids but not in the sister family Mycetophilidae [25,34,35]. Interestingly, Cecidomyiidae species also exhibit paternal genome elimination and X chromosome elimination as a means of sex determination [23,36]. However, if Haig’s hypothesis is correct, GRCs, paternal genome elimination, and X chromosome elimination as a means of sex determination evolved independently in these 2 clades. There is recent evidence suggesting that the X chromosomes in Cecidomyiidae and Sciaridae evolved independently [37], but besides this, how the reproduction systems in both the Cecidomyiidae and Sciaridae evolved remains a mystery, and very little empirical work has been done on this topic in either clade.

We conduct the first genomic analysis of GRCs in Diptera, with the goal of exploring the origin, evolution, and composition of these chromosomes in Sciaridae. GRCs in Sciaridae are historically referred to as L chromosomes; however, we refer to them as GRCs in this paper to more easily facilitate comparison with GRCs in other lineages. We can imagine a few different hypotheses for the origins of GRCs in Sciaridae, following what is known about their distribution and the genetic systems in Sciaridae and Cecidomyiidae. First, as suggested by Haig [30], GRC evolution may be independent in Sciaridae and Cecidomyiidae, with the GRCs in fungus gnats evolving in the common ancestor of Sciaridae. In this case, the GRCs likely exhibit homology to the chromosome they evolved from (most likely the X chromosome according to Haig’s hypothesis) as they would have evolved in the last 50 million years according to estimates of the age of Sciaridae [38–41]. Alternatively, GRCs may have evolved in the common ancestor of Cecidomyiidae and Sciaridae and been subsequently lost in other gnat species (specifically Mycetophilidae, which is the only closely related gnat family in which the absence of GRCs has been noted in several species [34,35]). In this case, the GRCs would be substantially older and less likely to have high similarity to chromosomes in the core genome of Sciarids. Finally, GRCs may have evolved through other means, for instance, introgression. This has been found for B chromosomes, specifically the B chromosome in the jewel wasp *Nasonia vitripennis*. This chromosome, which is a selfish chromosome that affects sex determination in its host, was found to arise through a hybridization event with a *Trichomalopsis* wasp [42].

To disentangle the origins of GRCs in Sciaridae, we sequenced germline and somatic tissue from *B*. *coprophila* and were able to unambiguously identify GRC scaffolds in a single genome assembly generated from both tissue types by comparing scaffold coverage and k-mer distributions between the 2 sequence types (with the idea that GRC sequences will be present in the germline but not in the soma). We also use the recently published reference genome for *B*. *coprophila* [43], which was produced from male early embryos after GRC elimination (i.e., is not expected to contain GRC sequence) to perform downstream analyses to compare the gene-content between GRCs, autosomes and the X chromosome in *B*. *coprophila*. We find that the 2 GRCs are gene rich and carry many homologs to the core genome. Contrary to Haig’s hypothesis [30], we do not find that the GRCs are derived from the X chromosome, rather, we find GRC homologs throughout the genome that are highly divergent from the GRC copy. Remarkably, phylogenomic analyses suggest that the GRC genes in *B*. *coprophila* are often more closely related to genes in the core genome of Cecidomyiids (which also carry GRCs) than Sciarids. A possible explanation for this unexpected result is that the GRCs in *B*. *coprophila* arose through a hybridization event between an ancient Sciarid and Cecidomyiid, with the GRCs containing genes originating from the Cecidomyiid ancestor. Our study raises questions about how this allopolyploidization may have occurred, why the GRCs were retained over time in Sciaridae, how they became restricted to the germline, and whether the GRCs do form a homologous chromosome pair. This study provides a foundation for the study of GRCs in Sciaridae, an understudied lineage with regards to GRCs, with great potential given the rich body of molecular and cytological research in Sciaridae [26,44,45]. Furthermore, fungus gnats are a broadly distributed species easy to collect and rear in the laboratory, allowing for future studies of function and diversity of GRCs in the family. This study also adds to the recent genomic studies on germline-restricted DNA in animals, suggesting that germline-restricted DNA often contains numerous protein coding genes [10–13].

## Results

One consequence of the unconventional genetic system in *B*. *coprophila* is that male somatic and germline cells have a different chromosome constitution. They differ in the presence of GRCs, but also in the frequency of the X chromosome (2 are present in germline cells, but only one is present in somatic cells) (**Fig 1A and 1B**). We used these differences in chromosome constitution to identify the GRCs in *B*. *coprophila* and to distinguish the X chromosome from autosomes. We sequenced adult male germline and somatic tissue and generated a genome assembly from both the germline and somatic sequence libraries (see **Materials and methods** and **S2 Text** for assembly information). The genome assembly is of a comparable size to microspectrophotometry estimates for the genome of *B*. *coprophila* [46] (**Table 1**).

**Table 1:** Size and gene content of autosomes (all 3 autosomes combined), X chromosome, and GRCs identified through k-mer and coverage differences between the somatic and germline tissue.

	Size (Mb)		Gene number	
	Expected [46]	This study	Urban and colleagues [43]	This study
Whole genome	362	398	23,117	41,418
Autosomes	225	162.4	18,254	17,802
X	49	52.9	4,863	4,277
GRC	88	154.1	NA	15,812
Unclassified	NA	28.2		3,527

Chromosome sizes are compared to microspectrophotometry estimates for *B*. *coprophila* [46], and gene number is compared to the reference genome assembly [43], which is not expected to contain GRC genes. See **S2 Table** for assembly statistics and **S3 Table** for more information on unclassified contigs.

GRC, germline-restricted chromosome.

### *Bradysia coprophila* GRCs are large and gene rich

In order to identify GRCs in our genome assembly, we utilized coverage differences and differences in k-mer profiles between the somatic and germline tissue sequencing libraries. We used the k-mer profiles to extract GRC, X-linked, and autosomal k-mers that we mapped to the assembly (**Fig 1D**). A k-mer score for each scaffold was then calculated as the number of mapped k-mer from the chromosome type with the most k-mers mapping to that scaffold divided by the length of the scaffold. We identified scaffolds that have a much higher coverage in germline tissue than somatic tissue (log_2_ germline/soma coverage ratio >0.5) (**Fig 1C**) and a high proportion (k-mer score >0.8) of GRC-specific 27-mers mapping to the scaffold (**Fig 1D**; see **Materials and methods** and **S2 Fig** for details). We used a conservative approach, assigning GRC scaffolds only if both methods agreed on the assignment. Through this method, we were also able to identify regions that belonged to the X chromosome or autosomes. However, due to somatic contamination of the germline tissue sequencing library during the tissue dissection procedure, the coverage difference and k-mer frequencies of X chromosome and autosomal sequences were less pronounced between the somatic and germline tissue libraries than expected. Through both the coverage and k-mer assignment of chromosomes, we identified 162.4 Mb of autosomal sequence, 52.9 Mb of X chromosome sequence, and 154.1 Mb of GRC sequence (**Table 1**). The 28.2 Mb of sequence that we were unable to classify (**Table 1**) represent cases when the 2 methods (coverage and k-mer-based) did not agree on chromosome assignment (10Mb) or cases where the k-mer identification score was not high enough to classify the scaffold using this method (18.2 Mb) (see **S3 Table**). Overall, the low level of conflicting assignments indicates high agreement of the 2 approaches. Our chromosome sizes are comparable to microspectrophotometry estimates for *B*. *coprophila* [46], with the exception of the GRC size, which is approximately double the size we would expect. The microspectrophotometry estimate of GRC size assumes that the 2 GRCs in sperm are homologous (i.e., the estimated size is half the total size attributed to the GRCs). Therefore, the larger GRC size in our genome assembly may indicate that the 2 GRCs have been (at least partially) assembled separately or that there is population polymorphism with the pooled sample we sequenced carrying 2 distinct GRCs. We explore this observation below.

We annotated 41,418 genes in our *B*. *coprophila* genome assembly: 17,802 on the autosomes, 4,277 on the X chromosome, and 15,812 on the GRCs (**Table 1**). The number of genes that we annotated on the autosomes and X chromosome are comparable to the recently published reference genome for *B*. *coprophila* [43] (**Table 1**), which did not contain GRC sequence.

### GRCs have homologs throughout the genome

To better understand the origins of the GRCs in Sciaridae, we conducted a reciprocal blast search with the amino acid sequence of all annotated genes with the goal of understanding whether the GRCs have homologs primarily on one chromosome in the genome (indicating their likely origin) and whether the divergence level of homologs can give us an idea of whether gene traffic has occurred from the core chromosomes to the GRCs after their formation. We also conducted a collinearity analysis to identify larger homologous blocks in the genome, in which we identified collinear blocks of 5 or more genes anchored to the reference assembly (for autosomal and X-linked genes) [43] or an assembly we generated with long-read data from male germline tissue (for GRC genes, see **S2 Text** for methods). This allowed us to increase the continuity of our assembly and to anchor genes within our assembly to known chromosomes (autosomes A-II, A-III, A-IV, and the X chromosome) in the reference genome. Overall, we wanted to gain a better understanding using the number, chromosomal location, and divergence level of homologs in the genome, of the events which led to the current genomic composition of the GRCs. Additionally, we wanted to determine whether GRC-GRC reciprocal blast hits are prevalent in the genome assembly, which would suggest that we sequenced 2 distinct GRCs and explain why the size of the GRCs was larger than expected.

**Fig 2:**
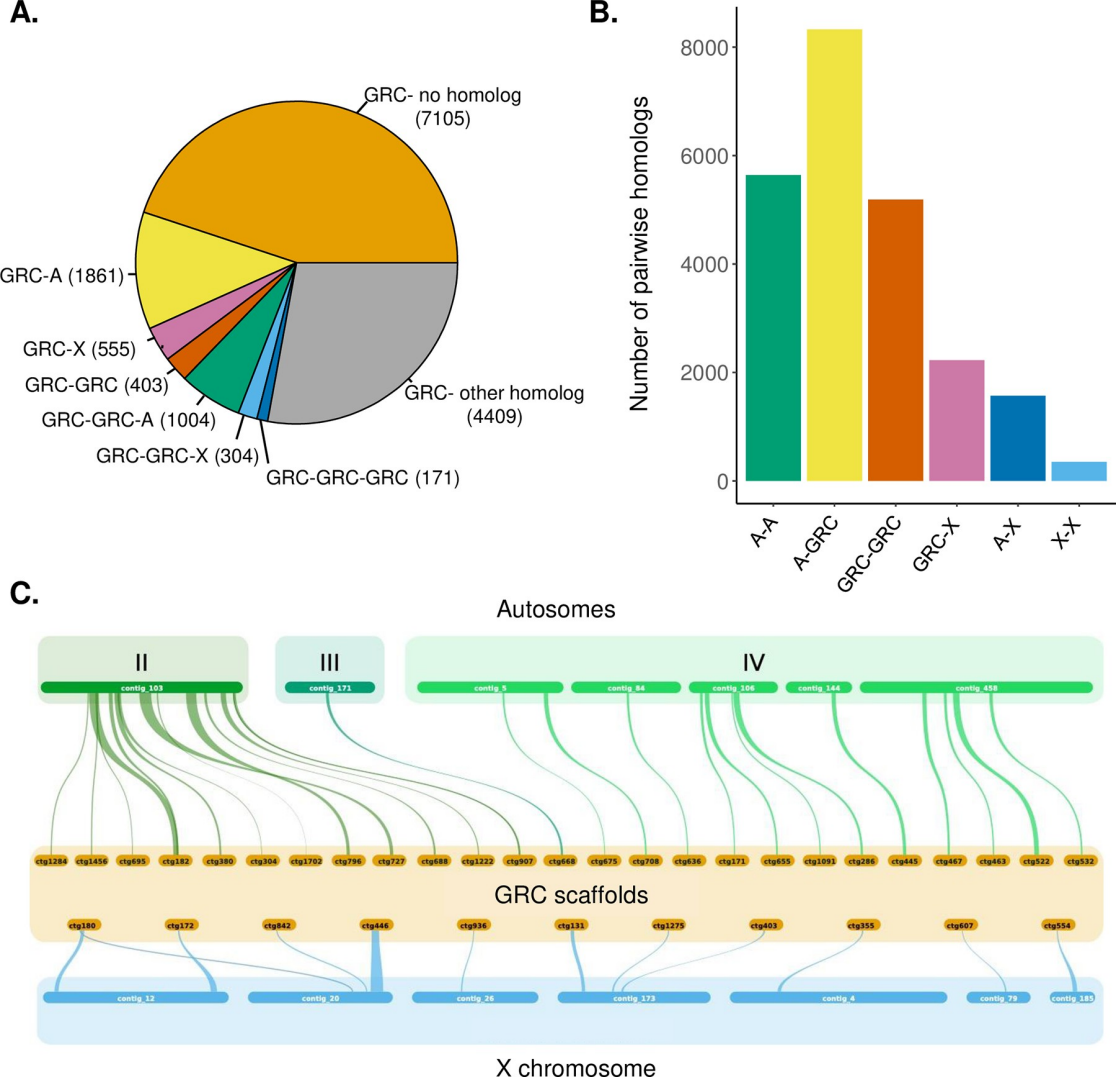
Many GRC genes have divergent homologs distributed throughout the core genome. **(A)** Number of GRC genes with no homologs, 1 homolog (and the location of that homolog), 2 homologs, or more homologs in the *B*. *coprophila* genome identified through a reciprocal blast analysis. Homologs in the GRC-other category either have multiple homologs (i.e., homologs with more than 4 genes with reciprocal blast hits to each other) or homologs with some genes on unclassified scaffolds. **(B)** Number of homolog pairs in the *B*. *coprophila* genome and chromosomal location of homologs. **(C)** Collinear blocks found between GRC scaffolds (orange) and scaffolds anchored to the X chromosome (blue) or individual autosomes (A-II, A-III, or A-IV; shades of green). Note that there is variation in the reference assembly in the proportion of scaffolds that are anchored to each chromosome (**S4 Table**). Location of data used to generate Fig 2A and 2B is specified in **S1 Table**. GRC, germline-restricted chromosome.

We looked for pairwise reciprocal blast hits in our genome assembly as well as identifying larger networks of genes that all had reciprocal blast hits to each other. From this analysis, we found that the GRC genes had many homologs located throughout the genome. We identified homologs in the genome for 55% (8707) of the 15,812 genes located on the GRCs, with 38.8% of these having exactly one homolog and the majority having multiple homologs in the genome (**Fig 2A**). We did not identify a homolog for the remaining 7,105 genes; however, the majority of homologs that we identified were unexpectedly divergent (approximately 45% amino acid similarity) (**S3 Fig**); therefore, there is a chance that some of these genes do have extremely divergent homologs within the genome assembly which we were unable to identify with the cutoff values used in our homology analysis (min 40% amino acid identity). Of the homolog pairs we identified (**Fig 2B**), 67% contained at least 1 GRC gene. We also identified homologs on all 3 autosomes and the X chromosome. The number of GRC-X chromosome homologs were slightly enriched compared to GRC-autosomal homologs given the number of genes on each of these chromosome types (Fisher exact test: odds ratio = 1.1125, 95% CI = 1.058 to 1.1844, *p* = 0.00023). However, the large number of homologs we identified on autosomes, and the fact that the divergence of the X-GRC homologs is similar to the divergence of A-GRC homologs (**S3 Fig**), suggests that the enrichment of X-GRC homologs does not likely reflect that the GRCs are derived from the X chromosome. We explore the origin of the GRCs in more detail below.

A collinearity analysis revealed 76 collinear blocks between the GRCs and autosomes, 13 collinear blocks between the GRCs and X chromosome, 23 collinear blocks in which both blocks were located on GRC scaffolds, and 5 collinear blocks in which both blocks were located on an autosome or the X chromosome. GRC-X chromosome collinear blocks made up a small proportion of blocks between the GRCs and core genome (14.6%) further suggesting that the GRCs are not likely derived from the X chromosome. We also anchored 42 blocks to individual chromosomes in the reference assembly and found that the GRCs are homologous to all 3 autosomes of *B*. *coprophila* (**Fig 2C**, **S4 Table**). Overall, our results suggest that the GRCs are not derived from the X chromosome, or from any other single chromosome, nor from a simple chromosomal rearrangement (e.g., fusion of a chromosomal arm and X chromosome). Intriguingly, our results also suggest that the homologs identified between the GRCs and core genome are older than we might expect if the GRCs evolved in the common ancestor of Sciaridae. We investigate the timing of evolution of the GRCs in more detail below.

### The 2 GRCs are divergent and show different sequencing coverage

**Fig 3:**
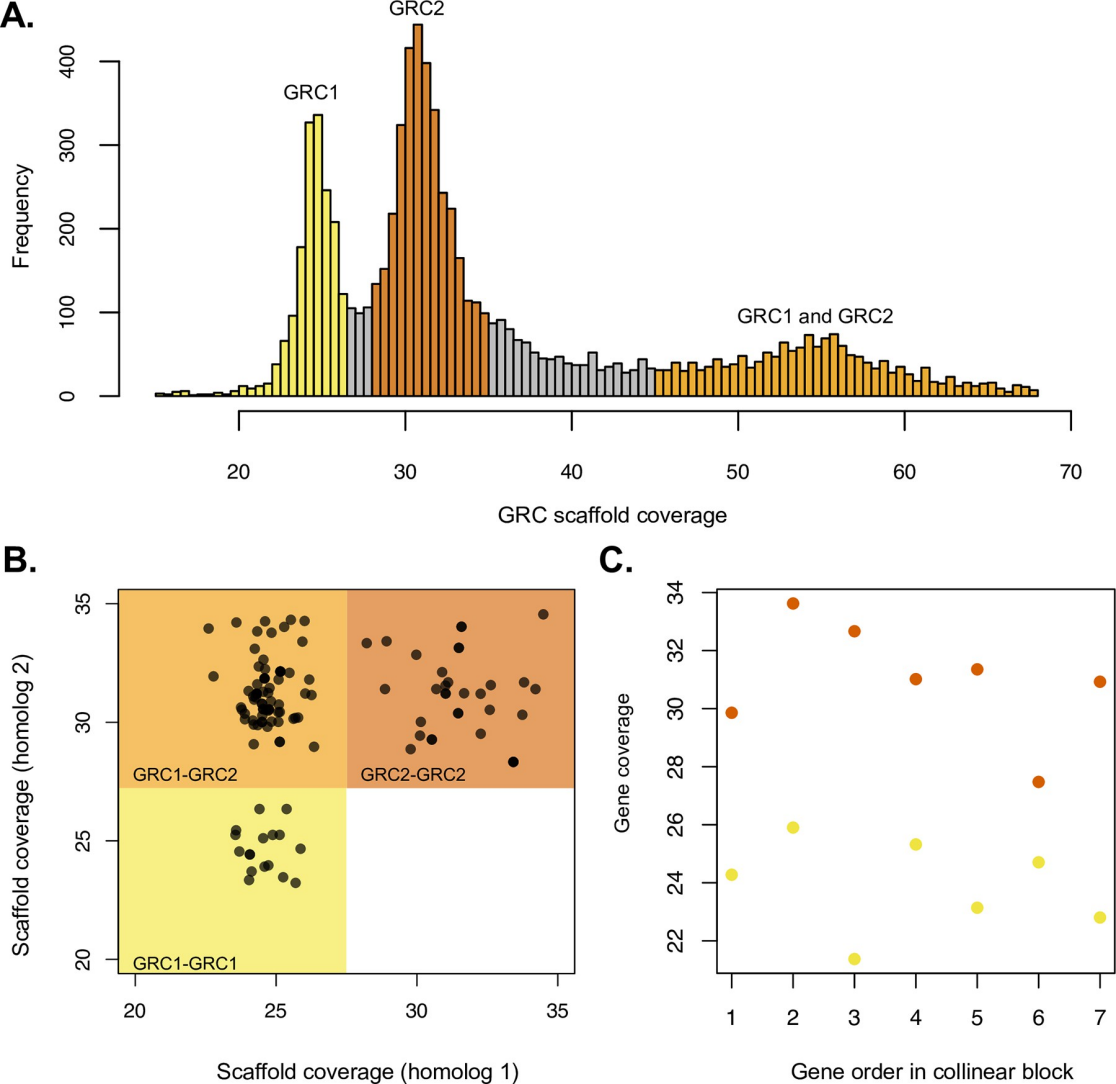
Coverage differences between the 2 divergent GRCs. **(A)** Histogram showing the coverage of scaffolds (up to 70×) assigned to the GRCs based on k-mer and coverage methods. The histogram shows 3 distinct peaks, 1 at 24.7× (yellow), 1 at 31.0× (dark orange), which represent the scaffolds belonging to the 2 GRCs (GRC1 and GRC2, respectively). The peak at approximately 55× (light orange) represents GRC scaffolds that the 2 GRCs have in common. **(B)** Scaffold coverage of 128 GRC-GRC homologs. Although the majority of homologs have one gene on GRC1 and the other gene on GRC2 (74, in light orange box), a substantial number of homologs also have both genes on GRC1 (20, yellow box), or both genes on GRC2 (34, dark orange box). **(C)** An example of a GRC-GRC collinear block in which one block is located on GRC1 (yellow) and therefore contains genes with a coverage between 20× and 27.5× and the other block is located on GRC2 (dark orange) and therefore contains genes with a coverage from 27.5× to 35×. Examples of GRC-GRC collinear blocks in which both blocks are located on the same GRC are in **S5 Fig**. Location of data used to generate Fig 3A–3C is specified in **S1 Table**. GRC, germline-restricted chromosome.

We wanted to explore the reason behind 2 unexpected observations: (1) that the size of sequence in our assembly attributed to the GRCs was much larger than the expected size of one GRC; and (2) that we identified a multitude of GRC-GRC homologs and GRC-GRC collinear blocks in our assembly. Therefore, we looked at the coverage of GRC scaffolds, to determine whether there was any evidence that we assembled 2 distinct GRCs. We analyzed the coverage of scaffolds assigned to the GRCs, the scaffold coverage of homologs in which both copies are on the GRCs, and the coverage of collinear blocks where both blocks are located on the GRCs. We found that the GRC scaffold coverage histogram has 3 peaks, 1 at 24.7×, 1 at 31.0×, and 1 at 55× (**Fig 3A**). As the third peak is approximately the sum of the mean coverages of the first 2 peaks, this likely indicates that we did indeed assemble 2 distinct GRCs, and the 2 lower coverage peaks represent scaffolds present only on 1 GRC (GRC1 = scaffolds at approximately 24.7× coverage, GRC2 = scaffolds at approximately 31.0× coverage). Scaffolds in the highest coverage peak (approximately 55× coverage) are likely scaffolds present on both GRCs, although these represent a much smaller proportion of GRC sequence compared to scaffolds only located on 1 GRC.

Next, we wanted to explore homology between the 2 GRCs by exploring whether the multitude of GRC-GRC homologs are an indication of distant homology between the 2 GRCs. To do this we assigned scaffolds to GRC1 or GRC2 by taking all GRC scaffolds with a coverage between 18× and 35× and a length greater than 5,000 bp and fitting 2 curves to this region of the histogram. We then assigned scaffolds to GRC1 or GRC2 by comparing the likelihood that they belong to one peak of the histogram versus the other (**S4 Fig**). Using this method, we assigned 43 Mb of the assembly to GRC1 and 63Mb to GRC2. We then determined whether GRC-GRC homologs had 1 copy on each GRC. We found that, although for the majority of GRC-GRC homologs we were able to assign (74/128), one gene was on GRC1 and the other on GRC2 (**Fig 3B**, light orange box), there were also a substantial number of GRC-GRC homologs where both genes were on GRC1 (20/128) or GRC2 (34/128) (**Fig 3B**), such that the enrichment of GRC1-GRC2 homologs was not statistically different from the assumption that homologs are randomly distributed in these 3 categories (i.e., a one to one ratio of GRC1-GRC2 homologs, and homologs only on one GRC; Fisher exact test, *p* = 0.26, odds ratio = 0.73, 95% CI = 0.43 to 1.23)). The majority of GRC1-GRC2 homologs observed is suggestive that the 2 GRCs do share homology, even on the scaffolds that assembled separately, but there are also many nonhomologous regions as many GRC homologs were only found on one GRC. In support of this idea, we also looked at whether collinear blocks in which both blocks were assigned to the GRCs had one block assigned to GRC1 and the other to GRC2 (**Fig 3C**). We found that 7/13 GRC-GRC collinear blocks which we were able to assign to GRC1 or GRC2 were of the GRC1-GRC2 type, however, we also found 5 blocks in which both blocks were on GRC2 and 1 block in which both blocks were on GRC1 (**S5 Fig**). Therefore, the 2 GRCs share some homologous regions, but also have some unique regions. However, due to the fragmented nature of our GRC assembly, we were unable to entirely resolve how the GRC scaffolds are physically placed, and future work is needed to determine where GRC1-GRC2 homologs are located on their respective chromosomes and where the GRC scaffolds at a higher coverage (55×) are physically placed on the GRCs.

### The GRC is old, and its evolutionary origins are obscure

In order to better understand how old the GRCs are, we reconstructed the phylogenetic placement of GRC genes in Sciaroidea (the superfamily which contains Sciaridae and Cecidomyiidae, which both carry GRCs, and several other gnat families that are not known to carry GRCs). We used a set of universal single-copy orthologs (BUSCO) identified in recently published draft genomes for 13 species within Sciaroidea and outgroup species (*Sylvicola fuscatus*) [37] (**S6 Fig**). We examined the phylogenetic placement of all BUSCO genes identified in *B*. *coprophila* which we were able to assign to a specific chromosome and for which we were able to identify the BUSCO gene in at least 80% of Sciaroidea species in the analysis (1,184 BUSCO IDs total) (**S5 Table** for chromosomal location of BUSCO genes). We first reconstructed a gene tree for every BUSCO ID and summarized the placement of genes located in the core genome and GRC genes.

**Fig 4:**
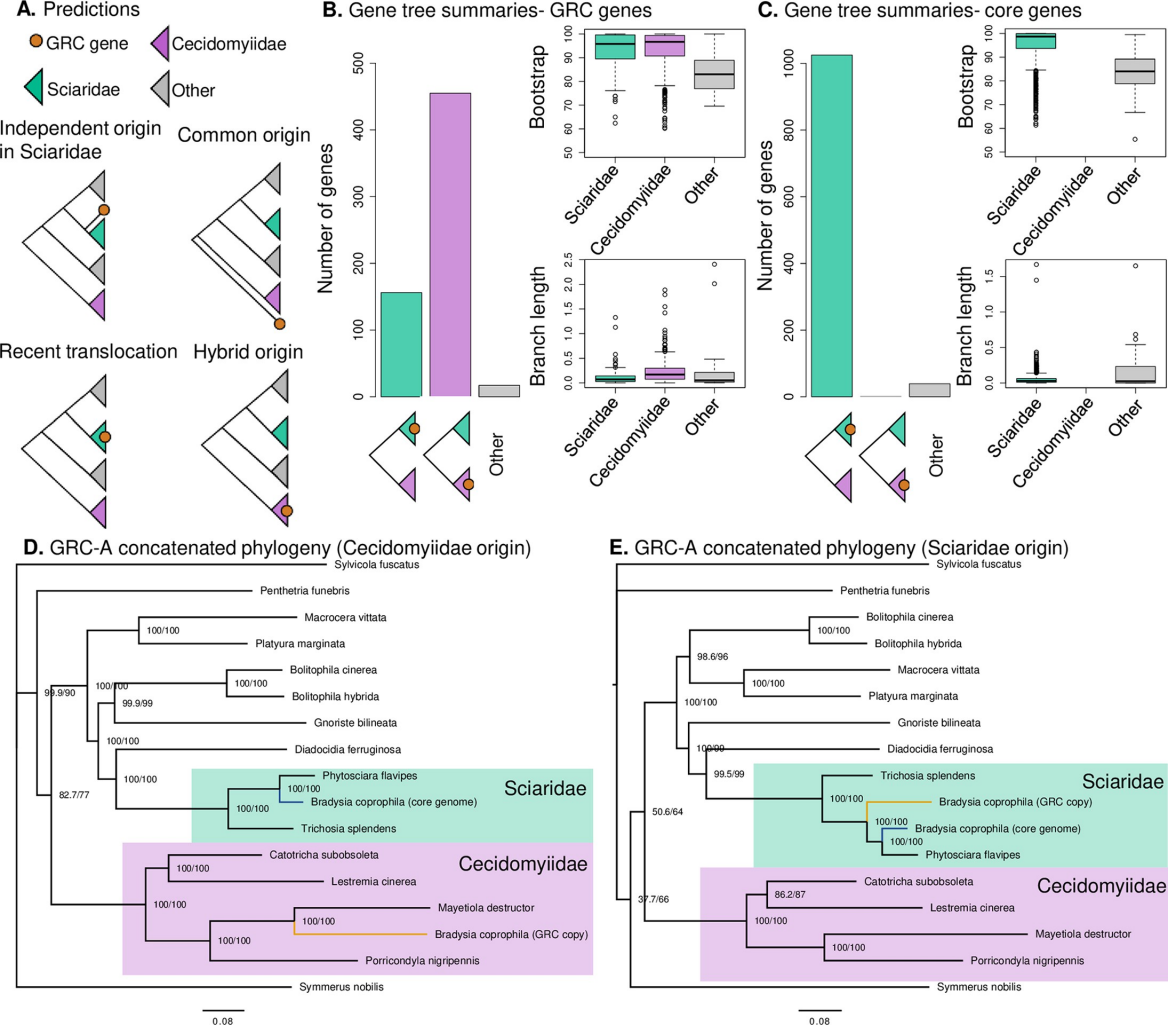
Phylogenetic analysis of conserved genes in *B*. *coprophila*. **(A)** Expected gene tree topologies given several hypothetical scenarios: evolution of the GRCs from a core chromosome at the root of Sciaridae, common evolutionary origin of GRCs in Sciaridae and Cecidomyiidae through a WGD event before the split of the lineages, evolution of the GRCs in Sciaridae via introgression from Cecidomyiidae, or recent traffic of genes from the core genome to the GRCs. **(B)** Summary of gene position in gene tree topologies for GRC BUSCO genes (627 total), with boxplots showing the bootstrap values for the GRC node and branch length of the GRC branch. The majority of genes fall within the Cecidomyiidae clade, with a sizable minority falling within the Sciaridae clade. **(C)** Summary of gene position in maximum likelihood gene tree topologies for core genome (autosomal or X chromosome) BUSCO genes (1,064 total), with boxplots showing the bootstrap values for the core gene node and branch length. The vast majority of core genome BUSCO genes fell within the Sciaridae clade as expected. See **S9 Fig** for examples of topologies of several BUSCO gene trees. **(D)** Maximum likelihood phylogeny generated from the concatenated amino acid alignments from 244 duplicated BUSCO genes in *B*. *coprophila* with 1 gene copy on either an autosome or the X chromosome and 1 copy on a GRC which fell within the Cecidomyiidae clade in gene tree phylogenies. **(E)** Maximum likelihood phylogeny generated from the concatenated amino acid alignments of 85 duplicated BUSCO genes in *B*. *coprophila* with 1 gene copy on either an autosome or the X chromosome and 1 copy on a GRC which fell within the Sciaridae clade in gene tree phylogenies. In the phylogenies, the values at nodes show the SH-aLRT/ultrafast bootstrap support. Location of data used to generate **Fig 4B–4E** is specified in **S1 Table**. GRC, germline-restricted chromosome; WGD, whole-genome duplication.

For GRC genes, we were interested in which hypothesis of GRC origin they supported (**Fig 4A**). We hypothesized that GRC genes would most likely have an intraspecific origin, where GRC genes were placed as the outgroup of the Sciaridae subtree (i.e., evolved in the common ancestor of Sciaridae). Another scenario is that the GRCs in Sciaridae and Cecidomyiidae share a common origin. Although it is generally considered that the GRCs in Sciaridae and Cecidomyiidae are independent [23,24], a single origin of GRCs in the common ancestor of the 2 clades with subsequent loss in other Bibionomorpha families has not been tested and could potentially explain the observation that GRC-core genome homologs in the genome are extremely divergent. Genes branching from the root of the common ancestor of the 2 clades support this scenario (however, such evolutionary scenario would likely lead to many unresolved trees due to saturation of sites as there would not be any closely related sequences in the phylogeny). We also explored support for the hypothesis that GRC genes could have arisen through introgression from another Sciaroidea family. Genes placed within a different Dipteran family than Sciaridae support this scenario. Finally, GRCs have likely been present in Sciaridae for more than 44 million years [38–40]; therefore, we might detect some GRC genes that were acquired after diversification of the Sciaridae family. Genes placed within the Sciaridae subtree support this scenario. However, this is not an evolutionary scenario explaining the origin of the GRCs, rather an indication about how dynamic the GRCs are.

As expected, BUSCO genes located on either an autosome or the X chromosome were nearly always located in the Sciaridae clade in phylogenies (1,028/1,067 genes or 96.3%) (**Fig 4C and 4E**, **S7 Fig**). Surprisingly, we found that 454 of the 627 GRC BUSCO gene trees (72.4%) support the origin of GRCs through introgression. All the genes supporting this origin are placed within the Cecidomyiidae family; specifically, the GRCs are most closely related to the hessian fly *Mayetiola destructor* (**Fig 4B and 4D**, **S7 Fig**). This placement is not caused by biases in amino acid composition (**S8 Fig**) and was supported by both bootstrap values and SH-like approximate likelihood ratio test score [47] (**Fig 4B**) and is therefore unlikely a methodological artifact.

The majority of the remaining genes (156, 24.8%) were monophyletic with all other Sciaridae genes. Of these, 74 genes had sequence data for all 3 Sciaridae species and allowed us to classify whether the GRC copy is an outgroup to other Sciaridae species or on an internal branch. Only 4 GRC genes (5.4% of those placed in Sciaridae, 0.6% of all GRC genes) supported the hypothesis that GRCs arose at the base of Sciaridae (i.e., were the outgroup to all other Sciarid species). The vast majority (70, 94.6%) of GRC genes in Sciaridae were placed on internal branches, suggestive of ongoing gene traffic onto the GRCs.

We reconstructed 2 concatenated phylogenies with the 340 BUSCO genes with 1 copy on the GRC and 1 copy on either an autosome or the X chromosome (i.e., GRC-A/X homologs), separating BUSCO IDs based on whether the GRC gene copy fell within the Cecidomyiidae clade or Sciaridae clade (**Fig 4D and 4E, S7 Fig**). The phylogenetic position of GRC sequences in *B*. *coprophila* is unexpected, but suggestive that the GRCs in *B*. *coprophila* might have arisen through an ancient hybridization event of a Sciaridae ancestor with a derived member of the Cecidomyiidae family. The support for the hybrid origin is surprising as the 2 families are extremely divergent, calling into question how the 2 genomes could be compatible [48]. However, phylogenetic analyses showed support for no other hypothesis. Assuming only a single origin of GRCs within Sciaridae, the GRC-linked genes that are placed within Sciaridae must represent postacquisition gene traffic (either translocations or duplications) from the core Sciaridae genome. This idea is consistent with the observation that the terminal branches of GRC genes within the Sciaridae family are significantly shorter compared to those within the Cecidomyiidae family (Mann–Whitney *p*-value < 0.0001; **S10 Fig**).

We also examined whether the phylogenetic position of GRC genes (either in the Cecidomyiidae or Sciaridae clade) is associated with whether the gene is located on GRC1 or GRC2. We found that both GRC chromosomes contained genes placed in Cecidomyiidae clade in phylogenies (GRC1 = 205 genes, GRC2 = 157 genes) and some genes located in Sciaridae clade in phylogenies (GRC1 = 24 genes, GRC2 = 97 genes), suggesting that both chromosomes likely originated through the same means (i.e., hybridization), followed by postacquisition gene traffic from the core genome. However, we found that GRC1 had more genes in the Cecidomyiidae clade in phylogenies compared to GRC2 (**S6 Table**) (Fisher exact test: odds ratio = 5.25, 95% CI = 3.16 to 9.02, *p* < 0.0001).

In further support of the hybrid origin hypothesis, we conducted a reciprocal blast search between the amino acid sequences of all GRC genes and the official gene set for *M*. *destructor*. We then extracted GRC genes that had a homolog in both the *M*. *destructor* genome and the *B*. *coprophila* core genome and compared the similarity of the homologs in each dataset. We found that most GRC genes had greater similarity to the *M*. *destructor* homolog rather than the *B*. *coprophila* core genome homolog (**S11 Fig**). Furthermore, similar to the phylogenetic analysis, we found that there was a smaller subset of genes that had greater similarity to the *B*. *coprophila* homolog and that these homologs overall had higher similarity values (likely representing recent gene traffic from the core genome).

## Discussion

### Perspectives on the origin of GRCs in Sciaridae

The results of this study raise many questions about how GRCs in Sciaroidea evolved (both in Cecidomyiidae and Sciaridae). Our study rejects the hypothesis that GRCs in Sciaridae arose from the X chromosome [30] and instead shows that many of the GRC genes in *B*. *coprophila* are more closely related to genes in the core genome of Cecidomyiids. However, there are still many questions remaining about how GRCs in Sciarids evolved, among them whether the GRCs arose from chromosomes in the core genome of a Cecidomyiid or from the GRCs. In the second case, the GRCs in both families would have a common origin. Although investigating the answer to this question would undoubtedly provide important insight into GRC evolution in Dipterans, we currently do not have GRC sequence data from any species within Cecidomyiidae, which would be needed to investigate this question. Such a dataset would be extremely useful to establish whether the GRCs in *B*. *coprophila* show greater similarity to the Cecidomyiid GRC genes or their autosomal counterparts.

While our results suggest introgression of GRC genes between the 2 clades, exactly how this introgression occurred is rather puzzling. There are a few examples where interspecies crosses gave rise to additional chromosomes with non-mendelian inheritance, such as the paternal sex ratio (PSR) chromosome in the parasitic wasp *Nasonia* [42,49]. The PSR chromosome is a B chromosome that interferes with sex determination in its wasp host and is thought to have evolved through hybridization with a parasitoid wasp in the genus *Trichomalopsis*. However, this B chromosome is a lot smaller (9 Mbp) compared to GRCs and the 2 species that hybridized have a much more recent common ancestor approximately 2.6 mya [50].

We roughly calibrated our phylogenetic tree by dated amber fossils from Bibionomorpha and estimated that the introgression happened 114 to 50 mya and between 37 and 101 mya after split of the 2 ancestors of Sciaridae and Cecidomyiidae (see **S3 Text** for details). This estimate gives us an approximate idea of when the GRCs introgressed into a Sciarid from a Cecidomyiid. We would like to note that this estimation is based on limited data and that there is also some debate as to the phylogenetic position of several of the Bibionomorpha families [24,38–41]. Therefore, although this is the best estimate we can at present make, it is possible that we overestimated the divergence between the Sciarid and Cecidomyiid ancestor when introgression occurred. However, our estimate suggests that the introgression event is of a much greater evolutionary distance compared to other reported introgressions. To our knowledge, the only natural hybridization event on this scale happened between 2 fern lineages after approximately 60 mya of divergence [51] (although animals of similar divergence have been successfully hybridized in the lab [52]). However, unlike Sciarid flies, the hybrid fern is incapable of sexual reproduction [51]. Hybridization based gene flow between very divergent lineages seems to be frequently associated with polyploidization (for example, in burrowing frogs [53] or *Arabidopsis* [54]). None of these examples is close enough to the pattern observed in our data; therefore, we propose a speculative model for the introgression via a single allopolyploidization event.

It is very unlikely the introgression would be a continuous hybridization process over such evolutionary distance. Therefore, the introgression probably took the form of a single hybridization event. The phylogenetic analysis placed nearly all the GRC genes in Cecidomyiidae closest to *M*. *destructor* (**Fig 4**); therefore, the hybridization event likely took place after differentiation of Cecidomyiidae. As GRCs and reproduction via paternal genome elimination is a common feature of the Cecidomyiidae family, the ancestor likely had both those features present during the hybridization event. The presence of the machinery for genome elimination in the *M*. *destructor* ancestor could have been the key for the allopolyploidization event to be successful. Perhaps, the Sciaridae subgenome retained its function as a core genome, while the Cecidomyiidae subgenome rearranged and became restricted to germline already in the first-generation hybrid, which might have facilitated overcoming reproductive barriers between the species. A previous phylogeny of 3 mitochondrial and 3 nuclear genome markers placed the 2 families far apart from each other with high confidence [24], indicating that the hybridization event likely happened between a male Cecidomyiidae and female Sciaridae ancestor. Cecidomyiidae have 2n = 8 core chromosomes and approximately 16 GRCs (although the GRC number varies), which are both substantially higher than the number of GRCs in Sciaridae, suggesting that the hybridized subgenome probably was subjected to substantial rearrangement. Although this scenario is very speculative, we believe it is the one best supported by our data. It would make the origin of the GRCs in *B*. *coprophila* through introgression from Cecidomyiidae, to our knowledge, the first example of cross-family allopolyploidization that led to sexually reproducing offspring.

We also found that phylogenetic position of GRC genes was associated with which GRC (i.e., GRC1 or GRC2) genes were located on, with GRC2 containing a larger number of genes that were located within the Sciaridae clade in phylogenies. Given that both GRCs had a substantial number of genes located in the Cecidomyiidae clade in phylogenies (**S6 Table**), we can conjecture that both GRCs likely arose in the same hybridization event. However, it is unclear why 1 GRC contains a higher proportion of Sciaridae genes, which we hypothesize originated via post hybridization gene traffic from the core Sciaridae chromosomes. Perhaps larger blocks of genes translocated from the core chromosomes to GRC2 compared to GRC1. We were able to assign more sequence to GRC2 than GRC1, indicating that this chromosome is likely larger. Further work with long-read/linked-read GRCs data may provide clues as to why this is the case, by elucidating the physical location of genes with different origins.

An alternative hypothesis to the origin of GRCs in Sciaridae through hybridization is that the GRCs arose through introgression via a different means such as horizontal gene transfer. Although horizontal gene transfer has been responsible for gene transfer between other lineages, including between insect species through either shared food sources or common parasitoids, etc. [55,56], the multitude of genes on the GRCs makes horizontal gene transfer an unlikely method by which these chromosomes have evolved, as the size of the GRCs is nearly the size of the entire *M*. *destructor* genome, and the GRCs contain more than 15,000 genes.

It is a striking coincidence that the presence of GRCs in Sciaroidea is associated with unconventional non-mendelian reproduction systems in both Cecidomyiidae and Sciaridae. Future studies will establish whether this is truly a coincidence or whether the unconventional transmission dynamics in both families somehow facilitates the evolution of GRCs or vice versa. For instance, the fact that the GRCs in Sciaridae are eliminated from somatic cells in much the same way as the X chromosome is eliminated for sex determination is suggestive either that (1) the GRCs have become established in the germline by manipulating the mechanism of sex determination or (2) that the system of sex determination in Sciaridae arose through manipulating the mechanism by which GRCs are eliminated from somatic cells. However, we need to learn much more about the genetic underpinnings of sex determination in these clades, and to establish the timing of the evolution of different parts of the chromosome system in these families to establish how or whether GRC evolution and the evolution of the unusual sex determination mechanism in Sciaridae and Cecidomyiidae are related.

### GRC features in Sciarids

It is interesting to consider how our results fit in with the larger body of cytological work on GRCs in Sciaridae. One unexpected finding is that there are 2 distinct GRCs in the pooled male germline tissue we sequenced. There are 2 possible reasons for this finding. This library was sequenced from a pooled sample of 95 male testes and as stated previously, all GRCs (normally 2) present in male germline cells are transmitted to sperm in *B*. *coprophila* [31]. Therefore, there may be 2 distinct GRCs because each germline cell (and sperm) in a male contains 2 distinct GRCs. If this is the case, the difference in GRC coverage for GRC1 and GRC2 is unexpected, as these 2 chromosomes should be at an equal frequency if they are both present in all germline cells. However, there is occasional variation in GRC number in *B*. *coprophila* germline cells [31]; therefore, the higher coverage of GRC2 versus GRC1 could indicate that this chromosome is systematically retained in germline cells more often than GRC1. Alternatively, the distinct GRCs may instead represent population polymorphism in GRCs, as we sequenced a pooled sample of males. In this case, the fact that GRC2 is at a higher frequency than GRC1 would indicate that, at least in the males we sequenced, this chromosome was more prevalent than GRC1.

In *B*. *coprophila*, the GRCs form a bivalent in female meiosis, suggesting that the GRCs are homologous [32]. Although it is unclear from our results whether the distinct GRCs are present in each germline cell or represent population polymorphism in GRCs, in either case we would not have expected the level of divergence we found in the GRCs if they pair during meiosis. This suggests either that the GRCs do not always (or potentially never) pair during female meiosis or that the distinct GRCs form a heteromorphic chromosome pair with little recombination when they do pair for meiosis. Furthermore, since we only sequenced male GRCs, and we know that GRCs do not undergo recombination/pairing in males, there is a possibility that the unusual transmission patterns of these chromosomes may result in sex specific GRCs which may explain observations of pairing in just females. Understanding more about which GRCs are present in different individuals in *B*. *coprophila*, and how these chromosomes are transmitted, will help us understand more about whether the 2 GRCs are present in all males and in females, whether the 2 GRCs ever pair during meiosis, and if so which portions of the GRCs undergo recombination, as we would not expect that level of divergence between the GRCs if a significant portion of these chromosomes recombined.

We do know that all GRCs in Sciaridae share one feature: restriction to the germline by elimination from somatic cells early in development. As we might expect that this feature is governed by a similar molecular mechanism in all Sciarids (and potentially also in Cecidomyiid GRCs), sequencing GRCs from a larger diversity of Sciarids can help us answer questions about how this occurs, by looking at what genes/genomic features are common between different GRCs (but not shared by the autosomes, for instance). In answering this question, the fact that the 2 GRCs we sequenced in this study were so divergent may come in handy, as there seems to be very few regions these chromosomes share. One other feature that Sciarid GRCs share is their unusual transmission patterns. In Sciaridae, all GRCs present in male germline cells are transmitted to offspring through sperm. Although this has only been established from a handful of species, these species are taxonomically distributed throughout the clade, suggesting that this may be a feature of all Sciarids [25,26,31,57]. In contrast, Cecidomyiids do not transmit GRCs through sperm as they are eliminated in the first meiotic division along with the paternally inherited chromosomes (through paternal genome elimination) [23,29]. Sequencing GRCs in a wider variety of species from both Sciaridae and Cecidomyiidae, and establishing transmission patterns of these chromosomes in more detail, may help to understand the underpinnings on why certain chromosomes are eliminated in the first meiotic division in these 2 clades.

An important caveat in this study is that although we have identified a multitude of genes located on the GRCs in *B*. *coprophila*, it is less clear whether all of these genes are functional. The GRCs in Sciaridae seem to have evolved in an unusual manner, and we would hypothesize that the when the GRCs originated through allopolyploidization, many genes on the GRCs (formerly genes in Cecidomyiids) would initially have been under relaxed selection. It seems possible that due to the unique origins of these chromosomes, some of the genes located on them may not be functional, and further work characterizing the role of GRC genes in development and reproduction of *B*. *coprophila* would be extremely useful. This work, however, was beyond the scope of our study. There was historically some debate about whether GRCs in Sciarids provided any sort of function, as these chromosomes are predominantly heterochromatic, except during a few stages during development and reproduction [31,58,59]. Since heterochromatin is gene poor, it was thought that few if any genes reside on the GRCs, similar to many B chromosomes, which often contain an excess of satellite DNA [49]. However, the sequence data presented here have revealed that there are many genes on the GRCs in *B*. *coprophila*, and future work can now elucidate when and where their transcription occurs and determine whether these chromosomes are necessary in *B*. *coprophila*.

### Comparison to songbird GRCs and other species with germline-restricted DNA

Several recent studies have sequenced germline-restricted DNA in different species that represent independent origins of this phenomenon. These studies, like ours, have generally found that germline-restricted DNA is more gene rich than expected [10,12,13,60]. It will be interesting to explore whether germline-restricted DNA in Sciarids, like in lampreys, nematodes, and songbirds, is also enriched in genes functioning in reproduction/germline function [10–13]. If so, an important question is whether these genes were originally present on the GRCs, or whether they were transferred to the GRCs after their origin (e.g., genes that fall within the Sciaridae clade in phylogenetic analyses). There is some experimental evidence that Sciarid GRCs may play a role in reproduction, specifically in sex determination, as it was found that a lab line of *Bradysia impatiens*, which lost the GRCs, also lost the ability to produce female offspring and thus later died out as a line completely [32]. Although this evidence is somewhat anecdotal, it is suggestive that like germline-restricted DNA in other lineages, GRCs in Sciarids may also play an important role in reproduction.

Songbirds are the only other lineage with GRCs in which the content of these chromosomes have been studied in detail [7,13,20,61]. Songbirds have a single GRC that is maternally transmitted and that seems to be present in all songbird species (i.e., has one origin within songbirds) [17,20]. In this lineage, most of the genomic work has been conducted on zebra finches [13]. In zebra finches, several hundred GRC genes have been identified, which have been transferred onto the GRC in stages since its origin [13]. This is in contrast to what we found for the GRCs in *B*. *coprophila*, in which it seems that most of the GRC genes originated in one time period (when the hybridization occurred), followed by some gene transfer to the GRCs more recently (GRC genes which fall within the Sciaridae in phylogenies). However, in both lineages it appears that there is some transfer of genes onto the GRCs over time from the core genome, perhaps reflecting that these chromosomes are acquiring genes related to germline function/reproduction over time [13]. It seems unlikely that the GRCs in songbirds and Sciarids evolved through the same process. In songbirds, it was suggested that the GRC evolved from a B chromosome which was restricted to the germline and domesticated over time [18]. For Sciarids, the GRCs likely arose through an ancient hybridization with a Cecidomyiid. However, in both lineages, GRCs may have been domesticated as a germline tissue specific chromosome over time. More work on the origins and function of GRCs in both lineages will provide answers to these questions.

## Concluding remarks

*B*. *coprophila* has a fascinating chromosome inheritance system, which displays several examples of non-mendelian transmission and contains 2 GRCs. Understanding more about how this system evolved can tell us about the evolution of alternative non-mendelian reproduction systems as well as about the evolution of GRCs and germline soma differentiation. Through sequencing the GRCs in the Sciarid *B*. *coprophila*, we have determined there are 2 distinct GRCs in this species that contain many protein coding genes. Perhaps the most intriguing finding from this study is that the GRCs in *B*. *coprophila* seem to have evolved through an ancient hybridization between a Cecidomyiid and a Sciarid. Future research is needed to establish exactly how this occurred, and whether it involved the core genome chromosomes of a Cecidomyiid, or the GRCs in this lineage, but this finding is the first example, to our knowledge, of a hybridization event between animals so divergent from each other that has had lasting evolutionary consequences. We speculate that the fact that the GRCs in *B*. *coprophila* are restricted to the germline (and these were the chromosomes which have a Cecidomyiid origin) is likely an important component to the longevity of this system, as otherwise we might expect negative effects due to chromosome pleiotropy in somatic cells.

Finally, our results add additional insight into the evolution of germline-restricted DNA. Studies on germline-restricted DNA in taxa with chromatin diminution (i.e., portions of chromosomes rather than whole chromosomes are restricted to the germline) often suggest that this system evolves to resolve germline/soma conflict over gene expression [12,62]. However, our results strongly suggest that the GRCs in *B*. *coprophila* evolved not as a means to resolve germline/soma conflict, but instead through allopolyploidization. The GRC in zebra finches, as well, may not have evolved as a means to resolve germline/soma conflict, but instead from a selfish B chromosome [18]. Investigating the evolution of GRCs in more lineages will help establish whether the catalyst for GRC evolution is different from chromatin diminution systems, but it seems that the origin of GRCs may often involve accessory chromosomes and therefore may evolve through genomic conflict. However, after germline-restricted DNA evolves, it seems to follow a similar evolutionary trajectory in both chromatin diminution and chromosome elimination systems, given that in both systems germline-restricted DNA are enriched for genes that function in germline maturation/function [10,12,13]. Understanding more about whether GRC genes are expressed in *B*. *coprophila*, and how and whether they have a germline related function, will provide additional insight into how different types of germline-restricted DNA are related, and whether GRCs in *B*. *coprophila* provide a similar function to other lineages with GRCs.

## Materials and methods

### Fly culture maintenance

*B*. *coprophila* lines used in this study have been maintained in the laboratory since the 1920s [44]. Most of the biological literature refers to this fly as *Sciara coprophila*, although the genus name was changed from *Sciara* to *Bradysia* some decades ago [63]. We refer to it here as *B*. *coprophila*, but *Sciara tilicola* (Loew, 1850), *Sciara amoena* (Winnertz, 1867) *Sciara coprophila* (Lintner, 1895), and *Bradysia tilicola* are all synonyms. Our *B*. *coprophila* cultures were obtained from the *Sciara* stock center at Brown University and kept at the University of Edinburgh since October 2017. We maintain colonies by transferring 1 female and 2 males to a glass vial (25 mm diameter × 95 mm) with bacteriological agar and allowing the offspring of the female to develop. During development, we add a mixture of mushroom powder, spinach powder, wheat straw powder and yeast to the vials 2 to 3 times a week until the larvae pupate.

### gDNA extractions and sequencing

We sequenced genomic DNA from somatic (heads) and germline (testes and sperm) tissue of 1- to 2-day-old adult males. We dissected males which had been put on ice in a vial (to slow down males) on a clean slide in a dish of ice under a dissecting scope. For the dissections, we used jewelers forceps to separate the head from the body and then placed the head in a 1.5-ml microcentrifuge vial on dry ice. We then placed a drop of sterile 1X PBS on the body of the male and used forceps and insect pins to slowly pull the claspers away from the body until the claspers and male reproductive tissue separated from the body. We severed the ejaculatory duct and placed the testes in a separate microcentrifuge tube. We collected males over several days and stored the samples at −80°C until DNA extractions, sequencing a pooled sample from the tissue from 95 males.

The DNA extraction protocol we used was a modified version of the DNeasy Blood and Tissue Kit (Qiagen, Germany) extraction procedure (see **S2 Text** for full protocol). We quantified DNA on a qubit fluorometer (v3) and sequenced the samples on the Illumina Novaseq S1 platform, generating PE data with 150 bp reads and 350 bp inserts through Edinburgh Genomics (Edinburgh, UK).

### Genome assembly and annotation

We generated a genome assembly with both the somatic and germline tissue short-read libraries (**S2 Table**). We also generated a genome assembly from long-read (PacBio Sequel 3.0) sequence data from germline tissue, but the short-read assembly produced a more complete genome assembly according to BUSCO gene assessments, so this assembly was used for gene annotation. We used the long-read assembly for the collinearity analysis to increase the continuity of GRC scaffolds (see **S2 Text** for details).

For the short-read libraries, we trimmed the raw reads with fastp with parameters —cut_by_quality5 —cut_by_quality3 —cut_window_size 4 —cut_mean_quality 20 [64] and used fastqc to investigate read quality (https://www.bioinformatics.babraham.ac.uk/projects/fastqc). We generated an initial assembly with CLC assembly cell using default settings (Qiagen- v 5.0.0), then used blobtools [65,66] to investigate contamination in the raw reads (see **S1 Fig** for blobplot), using bamfilter to retain reads which had a GC content between 0.14 and 0.51 and a coverage higher than 7 (which excluded Prokaryotic sequences identified as contaminants). We generated an assembly with spades [67] using the filtered reads and k-mer sizes of 21, 33, 55, and 77. We conducted a BUSCO analysis (version 4.0.2) [68] using the insecta database (insecta_odb10) to assess whether single-copy orthologs expected to be present in insect genomes are present in our draft genome. We then annotated the genome using the braker2 pipeline [69], aligning RNAseq reads from male and female germline tissue (see **S2 Text** for details) to the genome using Hisat2 (using default settings, v2.1.0) [70], and using RepeatModeler (v2.0.1 using default settings) [71] and RepeatMasker (v4.1.0) [72] with the RepeatModeler output and known insect repeats as the repeat library, and the settings -gff -gc 35 -xsmall -pa 32 -no_is -div 30 to mask the genome assembly.

### Identification of GRC scaffolds

We used a combination of 2 techniques to identify scaffolds belonging to the GRCs in our assembly. One technique employs coverage differences between the germline and somatic tissues to identify which chromosome a scaffold belongs to. Since the number and type of chromosomes differs between the somatic and germline tissue (**Fig 1B**), we expect autosomal scaffolds to have a log2 coverage ratio (germline/soma) of approximately −1 (i.e., at 2X the frequency in somatic tissue compared to germline), X-linked scaffolds to have a coverage difference of approximately 1, and GRC scaffolds to have very few reads mapping to them from the somatic library but a diploid coverage level in the germline tissue library (see **Fig 1** for a schematic of coverage expectations). We mapped the germline and somatic reads to the genome assembly with bwa mem (v0.7.17) using default settings, counted the number of reads from each library mapping to each scaffold, and computed the log2 ratio of germline read counts to soma read counts (+1 to ensure a noninfinite coverage ratio) [73]. Due to somatic contamination in the germline library, the coverage differences displayed the pattern we expected and we were able to distinguish autosomal and X linked scaffolds, but the autosomal and X chromosome scaffolds had less extreme coverage differences than expected (i.e., X chromosome scaffolds had a lower germline/soma log2 coverage difference than we expected as somatic contamination of the germline tissue library meant that the X chromosome reads were at a lower frequency than expected in this tissue and vice versa for autosomal scaffolds). Therefore, we manually determined the germline/soma cutoff ratios between each chromosome type. We labeled scaffolds with a coverage difference of >−1 to <−0.1 as autosomal, those with a coverage difference of >−0.1 to <0.5 as X-linked, and scaffolds with a coverage difference greater than 0.5 as GRC linked (**Fig 1C**).

The second technique we used to assign scaffolds to chromosomes utilizes differences in the frequency of k-mers in the trimmed sequencing reads of each library. We used the kat comp command (kat v 2.4.1) [74] to generate a 2D histogram comparing 27-mer composition between the germline and somatic libraries (**Fig 1D**). We manually inspected the 2D histogram and used the expected differences in the chromosome frequency in each tissue to assign k-mers with a frequency between 125 and 175 in the somatic library and between 80 and 140 in the germline library as autosomal, k-mers with a frequency between 50 and 100 in the somatic library and 60 and 100 in the germline library as X-linked, and k-mers with a frequency <5 in the somatic library and >10 in the germline library as belonging to the GRCs. We used a custom script to extract 27-mers and their coverages using kmc dump [75] and searched for exact matches to these k-mers in the assembled scaffolds using bwa mem (v0.7.17 with -k 27 -T 27 -a -c 5000 parameters) [73]. We had no prior expectations on the density of k-mers mapping to the scaffolds or the specificity of mapping; therefore, we generated a k-mer identification score defined as the number of k-mers mapping to the scaffold from the chromosome category with the most k-mers mapping to that scaffold divided by the length of the scaffold. Subsequent decision thresholds were based on manual inspections of distributions of scores (see **S2 Fig** for plots showing k-mer identification cores for each chromosome type). Scaffolds with a majority of autosomal k-mers mapping to them and a k-mer identification score greater than 0.4 were assigned as autosomal. Similarly X-linked scaffolds with a k-mer identification score greater than 0.4 were assigned as X-linked, and GRC scaffolds with a k-mer identification score greater than 0.8 were assigned as belonging to the GRCs (**S2 Fig**). We then compared the scaffolds assigned using the k-mer and coverage techniques. Only scaffolds that were assigned as the same chromosome type with both techniques were included in downstream analyses.

### Genome-wide homolog identification

We conducted an all-by-all blast search of annotated genes to identify gene homologs in our assembly using translated amino acid sequences (**Fig 2C**). First, we extracted transcripts for each gene with gffread (v0.11.7) [76] and used the longest transcript for each gene as the gene sequence. We identified homologs using reciprocal blast of translated genes with an e-value cutoff 1e^-10 and reciprocal hits that span at least 60% of both genes with a minimum of 40% similarity between reciprocal hits. We determined the number of homologs and their chromosomal locations and divergence levels with custom R scripts. We compared the number of X-GRC and A-GRC homologs to determine whether there was an enrichment in the number of X-GRC homologs with a Fisher exact test, comparing the number of unique genes from each chromosome (i.e., we did not include genes more than once if they had several homologs) with the number of genes in our annotation from each chromosome without homologs. For the collinearity analysis, we mapped GRC-linked genes to the long-read assembly (**S2 Text**), and autosomal and X linked genes to the reference assembly (NCBI accession: GCA_014529535.1 [43]) using blastn with an e-value cutoff of 1e^-10 (2.5.0+). Using the mapped set of genes and the amino acid reciprocal blast, we performed a collinearity analysis using MCScanX with default parameters (at least 5 colinear genes, genes must match the strand) [77]. Note that in the reference assembly, 20% to 46% of A-II, 8% to 19% of A-III, 37% to 52% of A-IV, and 93% to 100% of the X chromosomes are anchored (**S4 Table**) [43]. The synteny blocks between GRC scaffolds and individual anchored autosomal and X scaffolds, respectively, were visualized on **Fig 2C** using SynVisio (commit 4a4361f, [78]).

### Coverage analysis of GRC scaffolds and homologs

In order to explore why the size of the GRCs in our assembly was larger than we expected and why we observed so many GRC-GRC homologs, we explored the coverage of GRC scaffolds in the assembly. To do this, we used samtools to calculate the depth of each scaffold in the assembly [79] and extracted the coverage of GRC scaffolds in R. We first examined the histogram of coverages of GRC scaffolds and found that the vast majority of scaffolds (97.6%) had a coverage between 18× and 68×. There were 3 peaks in the coverage histogram, at approximately 25×, 30×, and 55× coverage. We reasoned that as 55× is approximately the sum of the coverage of the other 2 peaks, we likely have 2 distinct GRCs in our assembly and the scaffolds at 55× were those that the 2 GRCs have in common and therefore represented regions where the 2 GRCs assembled as one. The scaffolds at 25× or 30× were likely unique to one GRC; therefore, to assign scaffolds to GRC1 or GRC2, we considered scaffolds greater than 5,000 bp and with a coverage between 18× and 35×. We fit a mixture of univariate normals (normalmixEM function in the mixtools package in R, with k = 2, mu = 25 and 30 and lamba = 0.5, 0.5) to estimate normal distributions representing GRC1 and GRC2 from the coverage distribution. We assigned scaffolds to GRC1 or GRC2 by calculating the probability that they fit in both normal distributions and computed a log likelihood ratio. In the calculation, we assumed that each scaffold was coming from one or the other chromosome, and there were no multicopy scaffolds in the dataset as we filtered those with coverage >35×. We assigned any scaffold with a log likelihood ratio >−18 to GRC1 or GRC2.

We then looked at whether the GRC-GRC homologs and collinear blocks had one half on GRC1 and the other on GRC2, as we would expect if the 2 GRCs are largely homologous (but divergent enough to assemble separately). As we only assigned scaffolds to GRC1 or GRC2 if they met certain scaffold length/coverage criteria (see above), we assigned 128 GRC-GRC homologs and 13 GRC-GRC collinear blocks to GRC1 and GRC2. For the collinear blocks, we only made GRC1/GRC2 assignments on blocks in which we were able to assign more than 3 genes in the block to GRC1 or GRC2. We also examined the coverage of genes along each GRC-GRC collinear blocks using BEDtools coverage with settings -mean -a to compute the mean coverage across each annotated gene within the *B*. *coprophila* genome assembly (v2.26.0) [80] (**S5 Fig**).

### Phylogenetic analysis of the GRCs origin

We utilized draft genome assemblies for 14 Sciaroidea species and 2 species outside the Sciaroidea, most of which we obtained from Anderson and colleagues [37] with the exception of *M*. *destructor*, which we obtained from NCBI (accession: GCA_000149195.1). We conducted a BUSCO analysis (version 4.0.2) [68] using the insecta database (insecta_odb10) on each genome assembly, along with our *B*. *coprophila* assembly, to identify universal single-copy orthologs in each genome. We excluded the *Exechia fusca* genome from further analyses as this genome had a low proportion of complete BUSCO genes identified, indicating that the genome was likely of poor quality. We identified the chromosomal locations of each BUSCO gene identified in the *B*. *coprophila* assembly (**S5 Table**). We took the amino acid sequence of the BUSCO genes for *B*. *coprophila* (all copies) and the longest amino acid sequence for each BUSCO ID per species as the gene sequence in the genome assemblies from all other species (although note that most of the other Sciaroidea species had relatively low rates of gene duplication; see **S6 Fig**). We only retained BUSCO IDs in the analysis in which 80% of the species of interest had complete versions of the gene and for which we were able to determine the chromosomal location of all *B*. *coprophila* genes. This left 1,184 BUSCO IDs in our phylogenomic analysis.

We aligned the amino acid sequences with MAFFT using the L-INS-i method [81], setting either *S*. *fuscatus* or if that species was absent *Penthetria funebris* as the outgroup, according to recent phylogenetic studies [24,37]. We reconstructed a maximum likelihood phylogeny in IQtree for each BUSCO ID separately (as IDs contained different numbers of *B*. *coprophila* genes on different chromosomes depending on the ID) allowing IQtree to select the most appropriate substitution model and computing ultrafast bootstrap and SH-aLRT statistics for each node with 1,000 replicates [82–84]. We manually analyzed some phylogenies to determine patterns of GRC gene placement, then we analyzed the position of *B*. *coprophila* genes in all phylogenies using a custom script which summarized whether *B*. *coprophila* gene copies fell within the Sciaridae clade or Cecidomyiidae clade, as these were the 2 most common topologies. We also extracted information about the bootstrap value of the nearest node and branch length for *B*. *coprophila* genes in each phylogeny. For GRC genes that were placed in the Sciaridae clade, we also analyzed first whether gene sequences for all Sciaridae species were present in the gene tree, and second whether the GRC gene formed an outgroup to all other Sciaridae species (indicating that it potentially evolved in the common ancestor of Sciaridae) or whether the GRC gene was placed within the Sciaridae clade (indicating that it likely evolved through translocation from a core chromosome). As the placement of GRC genes in the Cecidomyiidae clade was unexpected given the evolutionary distance between these 2 clades, we also examined whether amino acid composition bias may have led to the GRC genes being placed in the Cecidomyiidae clade (i.e., due to long branch attraction). Therefore, we also generated a heatmap of the amino acid composition of all BUSCO genes that went into the phylogenetic analysis for all species.

We constructed concatenated phylogenies summarizing the most common position of GRC genes in phylogenetic analyses for the 340 BUSCO IDs for which we identified 2 genes in *B*. *coprophila*, one on the GRCs and one in the core genome (i.e., autosome or X chromosome). We separated the BUSCO IDs based on the phylogenetic placement of the GRC gene (i.e., either the Cecidomyiidae clade or Sciaridae), then reconstructed maximum likelihood phylogenies for both sets of genes separately in IQtree with 1,000 ultrafast bootstrap and SH-aLRT replicates [82–84]. For both the phylogeny in which GRC genes were placed within the Cecidomyiidae and the phylogeny in which GRC genes were placed within the Sciaridae, the protein model selected for the phylogeny was LG+F+R5, which is a free rate model that estimates amino acid frequencies from the data. We also identified which GRC chromosome (i.e., GRC1 versus GRC2) BUSCO genes were located on which were in either the Cecidomyiidae or Sciaridae clade in phylogenies. We conducted a Fisher exact test to determine whether phylogenetic position (Cecidomyiidae versus Sciaridae) was independent of GRC location (GRC1 versus GRC2).

### Homolog identification in M. destructor

Given the phylogenetic position of many GRC genes, we wanted to explore whether GRC genes share greater similarity with *M*. *destructor* homologs or homologs within the *B*. *coprophila* genome but on an autosome or the X chromosome. We therefore downloaded the amino acid sequences of the *M*. *destructor* official gene set from i5K (OGS1.0) and appended the amino acid sequences of the annotated GRC genes to this file. We then conducted a reciprocal blast search with the same parameters as we used for the homolog identification within the *B*. *coprophila* genome, filtering the reciprocal blast hits in the same way. We used a custom R script to extract GRC genes that had reciprocal blast hits in both the *M*. *destructor* and *B*. *coprophila* core genome. As some of these genes had more than one hit in one or the other genome, we took the reciprocal hit with the greatest identity within the genome to be the best blast hit for that genome. We then compared the similarity of reciprocal blast hit in the *M*. *destructor* genome to the similarity of the reciprocal blast hit for the same GRC gene in the *B*. *coprophila* core genome.

## Supporting information

S1 Text Detailed description of the chromosome inheritance system in *B*. *coprophila*. (PDF)

S2 Text Supporting information Methods. (PDF)

S3 Text Approximate dating of the timing of GRC evolution in *B*. *coprophila*. GRC, germline-restricted chromosome. (PDF)

S1 Table Location of data used to generate figures in main text and Supporting information figures. Scripts and analysis documentation describing how we generated figures/tables is available at https://github.com/RossLab/Bradysia-GRCs and at 10.5281/zenodo.5884857. All data to generate the figures within this manuscript are located at https://github.com/RossLab/Bradysia-GRCs/blob/master/tables/figure_data.tar.gz. The name of the data used to generate each figure is listed in this table.
(PDF)

S2 Table Summary statistics for the short-read and long-read assemblies used in this study. The short-read assembly was used for gene prediction as it was closer to the expected genome size compared to the long-read assembly and also was more complete according to BUSCO assessment.
(PDF)

S3 Table Size of unclassified scaffolds from k-mer and coverage identification methods. Chromosome assignment using the k-mer method is shown in the first column and the assignment with the coverage method is shown in the second column with the total size of unclassified sequence belonging to that category shown in the third column. In the k-mer assignment column, a “c” indicates cases where the majority k-mers from one chromosome mapped to that scaffold but the k-mer identification score (see **Fig 2A**) was too low to support a confident assignment with this method.
(PDF)

S4 Table Size and proportion of our genome assembly anchored to the *B*. *coprophila* reference genome [43] and the number of GRC homologs and number of GRC collinear blocks anchored to each chromosome in the reference assembly. GRC, germline-restricted chromosome. (PDF)

S5 Table Genomic location of universal single-copy orthologs (BUSCO) in *B*. *coprophila*. BUSCO assessment was conducted with the insecta_odb10 database. The genomic location of genes was identified with both coverage and k-mer identification techniques. Individual gene trees were examined for all categories of BUSCOs listed below (**Fig 4B and 4C, S7 Fig**) and concatenated phylogenies were generated for categories indicated with * (**Fig 4D and 4E**). BUSCO categories including unassigned genes (NA assignment) are excluded from this table.
(PDF)

S6 Table Phylogenetic placement of GRC BUSCO genes separated by whether the genes are on GRC1 or GRC2. Only GRC genes that fall within the Cecidomyiidae or Sciaridae clade are shown and only genes that have a scaffold coverage ranging from 18× to 35×. More genes on GRC1 fall within the Cecidomyiidae clade compared to genes on GRC2 (Fisher exact test: odds ratio = 5.25, 95% CI = 3.16 to 9.02, *p* < 0.0001). However, both GRC chromosomes contain some genes that are within the Cecidomyiidae clade in phylogenies. Numbers in parenthesis indicate genes that are likely present on that chromosome, but which we were unable to unambiguously classify to a specific GRC (i.e., genes with a c classification). GRC, germline-restricted chromosome.
(PDF)

S1 Fig Blobplot of unfiltered assembly generated from both germ and somatic libraries showing scaffold coverage versus scaffold GC (size of dot indicates scaffold size and color taxonomic assignment). Reads mapping to scaffolds with a GC content between 0.14 and 0.51 and a coverage higher than 7 were retained for the final assembly. Location of data used to generate this figure is specified in **S1 Table**.
(PDF)

S2 Fig Distributions of scores used in the k-mer identification technique. **(A)** Histogram of k-mer assignment scores for scaffolds of each chromosome type in the short-read assembly used throughout the manuscript. The score is defined as the number of k-mers with an exact match to the scaffold from the chromosome type with the majority of k-mers matching the scaffold, divided by the scaffold length. For GRC scaffolds (orange), we only assigned scaffolds with a score higher than 0.8 as GRC scaffolds, while for autosomal and X chromosome scaffolds (green and blue, respectively) we assigned scaffolds with a score higher than 0.4, as the GRC scaffolds had a more distinct k-mer profile (i.e., higher k-mer score) than autosomes and the X chromosome. **(B)** Histogram of k-mer assignment scores in the long-read assembly (see **S2 Text**). The scores are substantially lower, especially for differentiating autosomes and the X chromosome. This assembly was used only for anchoring GRC genes in longer blocks for the collinearity analysis. Location of data used to generate this figure is specified in **S1 Table**. GRC, germline-restricted chromosome.
(PDF)

S3 Fig Amino acid identity for reciprocal blast hits within the *B*. *coprophila* genome. Plots are separated by the location of genes involved in the blast hit, with **(A)** showing autosomal-autosomal homologs, **(B)** showing GRC-autosomal homologs, **(C)** showing GRC-GRC homologs, **(D)** showing GRC-X chromosome homologs, **(E)** showing autosomal-X chromosome homologs, and **(F)** showing X chromosome-X chromosome homologs. We set a threshold of 40% identity and genes covering at least 60% of each other to assign reciprocal blast hits, so we would to capture only hits that spanned most of the length of each gene. Location of data used to generate this figure is specified in **S1 Table**. GRC, germline-restricted chromosome.
(PDF)

S4 Fig Assignment of scaffolds to GRC1 or GRC2. Scaffolds longer than 5,000 bp, between 18× and 35× coverage and assigned to the GRCs were used to generate a density plot from which 2 normal curves were drawn (red curve = GRC1, blue curve = GRC2, dashed lines = means). We assigned each scaffold to GRC1 or GRC2 by taking the scaffold coverage and determining whether it was more likely to belong to GRC1 or GRC2. Location of data used to generate this figure is specified in **S1 Table**. GRC, germline-restricted chromosome.
(PDF)

S5 Fig Assignment of GRC-GRC collinear blocks to GRC1 or GRC2. **(A)** Barplot showing the number of the GRC-GRC collinear blocks we were able to assign to GRC1/GRC2. Blocks in which all but one gene in the same block were assigned to the same GRC are denoted with a c (i.e., GRC1-GRC2c). We were unable to assign several blocks either because one block did not have a consistent GRC assignment or because one block did not have enough genes on it which were assigned to one of the GRCs (because the scaffold lengths for the genes in these blocks were less than 5,000 bp). **(B–D)** Examples of gene coverages along a GRC1-GRC2 collinear block (B), a GRC1-GRC1 collinear block (C) and a GRC2-GRC2 collinear block (D). Location of data used to generate this figure is specified in **S1 Table**. GRC, germline-restricted chromosome.
(PDF)

S6 Fig Summary of universal single-copy orthologs (BUSCO) results for all Dipteran species in phylogenetic analyses. *Exechia fusca* was excluded from analyses as the proportion of complete BUSCOs was low (54%). In *B*. *coprophila*, 39.2% of the insect BUSCO genes were duplicated. Location of data used to generate this figure is specified in **S1 Table**.
(PDF)

S7 Fig Plots summarizing the phylogenetic position (i.e., Cecidomyiidae clade, Sciaridae clade, or other) of GRC (orange background) or core genome A genes (blue background- autosomal or X chromosome) for all categories of BUSCO IDs separately. For each BUSCO category, the barplot summarizes how many genes fall within the Sciaridae clade (teal), Cecidomyiidae clade (purple), or other (gray), the upper right boxplot shows the bootstrap values of the closest node for each gene within that category, and the bottom right boxplot shows the branch lengths for each gene within that category. Some categories are shown twice if they contain both GRC and core genes as these gene types were plotted separately. **Fig 4B and 4C** shows a summary of all GRC BUSCO genes and all core genome BUSCO genes, respectively. Location of data used to generate this figure is specified in **S1 Table**. GRC, germline-restricted chromosome.
(PDF)

S8 Fig Heatmap of amino acid composition of BUSCO genes used in phylogenetic analyses. Amino acid composition analysis did not show any potential bias that could cause long-branch attraction. For the genes used for the phylogenetic analysis we calculated the amino acid composition and used a heatmap to visualize the relative frequencies of individual amino acids. Overall, there is not much variation among the analyzed species. Furthermore, the composition of GRC genes (L-*Sciara_coprophilla*), clustered together with other Sciaridae genomes. The only *Sciara* genes with deviated aa compositions were those we were unable to classify (NA-*Sciara_coprophila*), which might be related to the same problems we experienced when we attempted to assign chromosomes (difficult structure). Location of data used to generate this figure is specified in **S1 Table**. Note: *Sciara_coprophila* is a synonym for *B*. *coprophila*. GRC, germline-restricted chromosome.
(PDF)

S9 Fig Examples of GRC gene trees with various topologies. **(A)** With 1 GRC copy rooted in Cecidomyiidae, **(B)** with 1 GRC copy rooted in Sciaridae, **(C)** with 2 GRC copies both in Cecidomyiidae, **(E)** with 2 GRC copies, 1 in Cecidomyiidae and the other in Sciaridae, **(D)** with 2 GRC copies both in Sciaridae. **(F)** GRCs unplaced (without significant nodes) or branching with a species from any other family. Location of data used to generate this figure is specified in **S1 Table**. GRC, germline-restricted chromosome.
(PDF)

S10 Fig Terminal branch length distribution of GRC genes. Branch length distribution of GRC copies of BUSCO genes plotted with respect to the phylogenetic position (at family level), means shown by dashed lines. Branch lengths of BUSCO genes on GRCs within Cecidomyiidae (violet) are significantly longer than branches found within Sciaridae (teal; *p*-value < 0.0001) suggesting the genes on GRCs found within Sciaridae might be due to gene duplications and translocations within Sciaridae after the GRCs were acquired. Location of data used to generate figure is specified in **S1 Table**. GRC, germline-restricted chromosome.
(PDF)

S11 Fig Comparison of GRC homolog amino acid identity to *M*. *destructor* or *B*. *coprophila* core genes. **(A)** Scatterplot of the amino acid identity for GRC genes that had a reciprocal blast hit to both the *B*. *coprophila* core genome (x-axis) and the *M*. *destructor* core genome (y-axis). The majority of genes had a greater similarity to the *M*. *destructor genome*. **(B)** For genes that had a greater similarity to the *B*. *coprophila* genome (teal background in A), a histogram of the homolog identity to the *B*. *coprophila* core genome gene, with **(C)** showing a histogram of the homolog identity to the *M*. *destructor* core genome gene for genes that had a greater similarity to the *M*. *destructor* genome (purple background in **A**). **(D)** Histogram showing the number of reciprocal blast hits for GRC genes to the *B*. *coprophila* core genome, the number of unique hits (i.e., taking only one hit for each GRC gene with the highest identity), the number of reciprocal blast hits for GRC genes to the *M*. *destructor* core genome, the number of unique hits, and the number of GRC genes which had a reciprocal blast hit in both the *M*. *destructor* and *B*. *coprophila* genome. The genes in the last category were used for plots A,B, and C. Note that comparisons of reciprocal blast hits between these 2 genomes should be taken with a grain of salt, as the *M*. *destructor* genome was not annotated in the exact same way as we annotated the *B*. *coprophila* genome. However, we would not expect this to substantially affect the patterns of homology shown above. Location of data used to generate this figure is specified in **S1 Table**. GRC, germline-restricted chromosome.
(PDF)
