# Supplementary material for: Gene-rich germline-restricted chromosomes in black-winged fungus gnats evolved through hybridization
Source: PLoS Biol. 2022 Feb 25;20(2):e3001559. doi: 10.1371/journal.pbio.3001559 (PMC8906591; doi:10.1371/journal.pbio.3001559)
Supplement: S1 Text — (PDF) [file pbio.3001559.s001.pdf]

Supplementary Information for, "Gene-rich germline-restricted chromosomes in black-winged fungus gnats evolved through hybridization."

Christina N. Hodson, Kamil S. Jaron, Susan Gerbi, Laura Ross

## S1 Text: Detailed description of the chromosome inheritance system in *Bradysia coprophila.*

The chromosome system in *B. coprophila*, and in sciarids generally, is unique in several ways including chromosome transmission patterns, sex determination, and the presence of GRCs (see **Fig 1A** for transmission patterns). All sciarids studied to date have a system of reproduction known as paternal genome elimination, where males only transmit maternally inherited chromosomes to offspring [1,2]. Paternal genome elimination has evolved independently in at least seven arthropod lineages, including the related gall gnat family Cecidomyiidae [3]. In all species with paternal genome elimination, meiosis occurs in a Mendelian manner in females (i.e. the chromosome transmitted to eggs is random with respect to parental origin), but in males meiosis is aberrant. In male meiosis in sciarids, there is a monopolar spindle in meiosis I. Maternally inherited chromosomes move towards the monopolar spindle, while paternally derived chromosomes move away from it and are discarded in a bud of cytoplasm [2]. Thus, only the maternal complement of chromosomes is transmitted to the sperm. This phenomenon in *B. coprophila* was the first recognised example of "imprinting", to our knowledge, by which the cell recognizes the maternal or paternal origin of a chromosome [4]. Interestingly, the GRCs always segregate with the maternal set of chromosomes. Therefore, GRCs (typically two in *B. coprophila*) are transmitted through sperm, regardless of whether they are of maternal or paternal origin [4]. This is one of the

only examples of chromosomes which seem to evade paternal genome elimination. In the second division of meiosis in *B. coprophila* there is a bipolar spindle, however there is a nondisjunction of the maternal X chromosome in this division such that only one sperm develops through male meiosis, which contains two X chromosomes. This sperm contains a haploid set of autosomes, typically two GRCs, and two X chromosomes [1,2]. There is some variation in the number of GRCs in each sperm, ranging from 0-4 in *B. coprophila* [4]. Variation in GRC number is thought to be due to nondisjunction events which can occur in early germ cell divisions, however, the majority of sperm (78%) carry two GRCs [4]. In female meiosis, the GRCs form a bivalent during meiosis, and one GRC segregates into each egg (i.e. meiosis is thought to be typical) [4].

As a result of the unconventional meiosis in male sciarids, *B. coprophila* zygotes typically carry a diploid set of autosomes, three X chromosomes (one inherited from their mother and two from their father), and three GRCs. All sciarids have XO sex chromosome system (i.e. males are XO and females are XX, and there is no Y chromosome), but sex is determined via X chromosome elimination from somatic cells early in development. In the 7-9th cleavage division, either one X chromosome (for females) or two X chromosomes (for males) are eliminated from somatic cells (eliminated chromosomes are always of paternal origin) [5]. Elimination occurs due to a failure of separation of the sister chromatid arms during mitosis, resulting in the chromosomes being left on the metaphase plate and not being incorporated into daughter nuclei [6]. It is thought that the number of X chromosomes eliminated is maternally controlled, since *B. coprophila* females are monogenic, and produce exclusively female or male progeny [7]. Females that produce female offspring carry a large inversion on the X chromosome that is always associated with female-producing females [2,8]. GRC elimination from somatic cells occurs in a remarkably similar manner, with the

exception that GRC elimination occurs in the 5-6 cleavage division and all GRCs are eliminated from somatic cells [5].

In germ cells, there is also an elimination of one X chromosome and typically one GRC. In this case, elimination occurs in a somewhat mysterious manner in early germ cell development, when one X chromosome and all but two GRCs are eliminated by being ejected from the germ cell through a cytoplasmic bud [9,10]. Therefore, early germ cells of both males and females in *B. coprophila* have the same chromosome constitution, with a diploid set of autosomes, X chromosomes, and two GRCs. This mechanism is also thought to regulate the number of GRCs and prevents their accumulation over time, as all but two GRCs are always eliminated from early germ cells.

Less is known about the mechanism of the chromosome system in other sciarid species, but across the family all species studied exhibit paternal genome elimination and X chromosome elimination early in development as the means of sex determination. Although only a handful of species have been studied in detail, evidence suggests that most, but not all sciarid species carry GRCs, with the number of GRCs ranging from 0-4 [2,11]. Two species, *Bradysia reynoldsi and Bradysia ocellaris,* in which GRCs are known to be absent are closely related to each other, suggesting that GRCs were likely lost in these species (i.e. they evolved from an ancestor with GRCs). Additionally, monogeny, or females that produce offspring of only one sex, is present in some, but not all species across Sciaridae [2]. There seem to be many transitions in this trait across Sciaridae, with some species being monogenic, some being digenic (i.e. females produce offspring of both sexes), and some species having a mix of these two types of females. Very little is known about the genetic underpinnings of this trait.

Overall, the evidence suggests that paternal genome elimination and X chromosome elimination as a means of sex determination evolved once in the common ancestor of Sciaridae. It is less clear how GRCs and monogeny evolved. It was originally suggested that the presence of GRCs and monogeny are related, as *Bradysia reynoldsi,* a species that lacks GRCs, is digenic (although note, *Bradysia ocellaris* which also lacks GRCs can be either monogenic or digenic). Additionally, a lab line of *Bradysia impatiens* that was bred to lose GRCs transitioned from monogenic to digenic reproduction [4]. However, these facts are anecdotal and there are also several species with digenic reproduction that do carry GRCs (reviewed in [2]).

Cecidomyiidae, gall gnats also in the Infraorder Bibionomorpha, have a similar reproduction system to Sciaridae, in that both families exhibit paternal genome elimination and X chromosome elimination as a means of sex determination, GRCs, and a mix of monogenic and digenic species [12]. However, cecidomyiid species have two X chromosomes (i.e. females are $X_1X_1X_2X_2$ and males are $X_1X_2OO$), and the factor that controls X chromosome elimination in offspring (in the cecidomyiid *M. destructor*) is associated with an inversion on an autosome (rather than the X chromosome in *B. coprophila*) [13]. Additionally, GRC characteristics are quite different in this family, with species containing many small GRCs, which are maternally transmitted and do not form bivalents during female meiosis.

**Supplementary References**


1. Metz CW. Chromosome behavior, inheritance and sex determination in *Sciara*. Am Nat. 1938;72: 485–520. doi:10.1086/280803

2. Gerbi SA. Unusual chromosome movements in sciarid flies. Results Probl Cell Differ. 1986;13: 71–104. doi:10.1007/978-3-540-39838-7_2

3. Gardner A, Ross L. Mating ecology explains patterns of genome elimination. Ecol Lett. 2014;17: 1602–12. doi:10.1111/ele.12383

4. Crouse H V, Brown A, Mumford BC. L-chromosome inheritance and the problem of chromosome “imprinting” in *Sciara* (Sciaridae, Diptera). Chromosoma. 1971;34: 324–339. doi:10.1007/bf00286156

5. Du Bois AM. Chromosome behavior during cleavage in the eggs of *Sciara coprophila* (Diptera) in the relation to the problem of sex determination. Zeitschrift für Zellforsch und Mikroskopische Anat. 1933;19: 595–614. doi:10.1007/BF00393361

6. de Saint Phalle B, Sullivan W. Incomplete sister chromatid separation is the mechanism of programmed chromosome elimination during early *Sciara coprophila* embryogenesis. Development. 1996;122: 3775–3784.

7. Metz CW, Schmuck LM. Unisexual progenies and the sex chromosome mechanism in *Sciara*. Proc Natl Acad Sci U S A. 1929;15: 863–866. doi:10.1073/pnas.15.12.863

8. Crouse H V., Gerbi SA, Liang CM, Magnus L, Mercer IM. Localization of ribosomal DNA within the proximal X heterochromatin of *Sciara coprophila* (Diptera, Sciaridae). Chromosoma. 1977;64: 305–318. doi:10.1007/BF00294938

9. Rieffel SM, Crouse H V. The elimination and differentiation of chromosomes in the germ line of *Sciara*. Chromosoma. 1966;19: 231–276. doi:10.1007/BF00326917

10. Perondini ALP, Ribeiro AF. Chromosome elimination in germ cells of *Sciara* embryos: Involvement of the nuclear envelope. Invertebr Reprod Dev. 1997;32: 131–141. doi:10.1080/07924259.1997.9672614

11. Hodson CN, Ross L. Evolutionary perspectives on germline-restricted chromosomes in flies (Diptera). Genome Biol Evol. 2021;13: 1–19. doi:10.1093/gbe/evab072

12. White MJD. Animal cytology and evolution. 3rd ed. Cambridge: Cambridge Univ Press; 1973.

13. Benatti TR, Valicente FH, Aggarwal R, Zhao C, Walling JG, Chen MS, et al. A neo-sex chromosome that drives postzygotic sex determination in the Hessian fly (*Mayetiola destructor*). Genetics. 2010;184: 769–777. doi:10.1534/genetics.109.108589
