# Supplementary material for: Gene-rich germline-restricted chromosomes in black-winged fungus gnats evolved through hybridization
Source: PLoS Biol. 2022 Feb 25;20(2):e3001559. doi: 10.1371/journal.pbio.3001559 (PMC8906591; doi:10.1371/journal.pbio.3001559)
Supplement: S2 Text — (PDF) [file pbio.3001559.s002.pdf]

S2 Text- Supplementary Methods

DNA extraction procedure

For gDNA extractions, for both short read and long read libraries we followed a similar protocol. All the centrifugation steps took place at 4°C and 13,000rpm, unless otherwise stated. Tissue samples were stored at -80°C until DNA extractions. Before extraction, we briefly froze the samples in liquid nitrogen and crushed the tissue with a micro-pestle. We then added 360µl of Cell Lysis Buffer (Qiagen) with 40µl of Proteinase K (20 mg/ml) (Qiagen), and incubated overnight in a shaking incubator at 55°C. We added 4µl of RNase A (100 mg/ml), mixed by inverting the sample tube, and incubated the sample for 1 hour at 37°C. We cooled the sample on ice for 5 minutes, then added 133 µl of Protein Precipitate Buffer (Qiagen), mixed by gently vortexing the sample and incubated on ice for 10 min. We then centrifuged for 15 min at 4°C, transferred the supernatant to a new tube containing 400µl isopropanol, and mixed by inversion. For the short read samples, we then incubated the sample overnight at -20°C, while for the long read samples, we incubated the sample for 10 min at room temperature. We then centrifuged the sample for 20 min, and discarded the supernatant by inverting the tube. We washed the DNA pellet twice with 300µl freshly prepared 70% EtOH, then centrifuged the sample for 20 min, and carefully removed the supernatant by pipetting. We air dried the DNA pellet for approximately 30 min, and resuspended the pellet in 60µl TE after it dried.

Long-read assembly

In addition to the short read sequencing data, we also extracted DNA (using the protocol above) from approximately 250 male testes to generate long read germline data. We sequenced the sample at Liverpool Genomics using a low input library prep procedure (i.e. no size selection) and PacBio Sequel 3.0 sequencing on 3 SMRT cells. We used red bean

(previously known as wtdbg2) with the parameters -L 1000 -x sq for the initial genome assembly (v2.5) [1], then polished the assembly three times with the long read library using minimap2 with parameters -c -x map-pb to map the long reads to the assembly (v2.17-r941) [2] and racon with parameter -u to polish the assembly (v1.4.10) [3]. We then polished the assembly twice with short read data (with only the germline library) using minimap2 and polishing with racon (see **Supplementary Table 1** for assembly statistics).

The long read assembly, compared to the short read assembly, showed two unfortunate problems: much lower mapping rates of GRC k-mers (**Supplementary Fig 2B**), and lower BUSCO score (BUSCO score: 93.6% complete BUSCOs vs. 98.3% for the short read assembly). We suspect the problems stemmed from high error profiles of long reads combined with high levels of homology across the genome hindering precise genome polishing and subsequently leading to frame shifts in gene models. This problem could be resolved in the future with newer sequencing approaches, such as HiFi reads with much smaller error rates, or Haplotagging. However, the long read genome assembly still featured much higher continuity (N50: 576,242 vs 18,920 for short-read assembly). Therefore, we used the short read assembly for annotation and gene level comparisons but used the long read assembly to link individual GRC genes found in the short read assembly for the collinearity analysis (**Fig 2C**).

RNAseq data generation

We generated 50bp paired end mRNA reads from germ tissue of male and female *B. coprophila* to help annotate the genome assembly. We generated three replicates for each sex composed of pooled dissected germ tissue from late larvae/early pupae from 400 individuals per replicate. We used the PureLink RNA Mini Kit (ThermoFisher Scientific) for RNA

extractions. We first added 50µl Trizol into each sample and crushed the tissue with a micropestle, then added 350µl of Trizol, briefly microcentrifuged the tubes, and transferred the supernatant to a clean tube. We added 80µl of BCP (1-Bromo-3-chloropropane), shook the sample by hand for 15sec and incubated on ice for 3 min. We then centrifuged the samples at 4°C for 15 min (13,000rpm for all centrifugation steps), transferred the supernatant to a new tube and added an equal volume of freshly prepared EtOH, mixing the tube by vortexing. We transferred the supernatant to a spin column and centrifuged the sample for 30sec. We discarded the flow through and added 350µl of Wash Buffer I to the sample. We centrifuged for 30 sec, then added 80ul of the DNase mixture (made of 8µl 10X DNase I reaction buffer, 10µl resuspend DNase I, and 62µl of RNase-free water) onto the membrane of the spin column. We incubated this mixture for 15 min at room temperature, then added 350µl of Wash Buffer I to the sample and centrifuged for 30sec. We then placed the spin cartridge into a new collection tube, added 500µl of Wash Buffer II, and centrifuged the sample for 30sec. We repeated this step once, then centrifuged the sample for an extra minute to dry the membrane. We placed the spin cartridge into a clean Eppendorf tube, added 30µl RNase-free water to the membrane, and incubated the sample for 1min. We sequenced the samples at Edinburgh Genomics, producing 50bp poly-A selected paired end reads on the NovaseqS1 platform.

References

1. Ruan J, Li H. Fast and accurate long-read assembly with wtdbg2. Nat Methods. 2020;17: 155–158. doi:10.1038/s41592-019-0669-3
2. Li H. Minimap2: Pairwise alignment for nucleotide sequences. Bioinformatics. 2018;34: 3094–3100. doi:10.1093/bioinformatics/bty191
3. Vaser R, Sović I, Nagarajan N, Šikić M. Fast and accurate de novo genome assembly

from long uncorrected reads. Genome Res. 2017;27: 737–746.

doi:10.1101/gr.214270.116
