# Supplementary material for: Gene-rich germline-restricted chromosomes in black-winged fungus gnats evolved through hybridization
Source: PLoS Biol. 2022 Feb 25;20(2):e3001559. doi: 10.1371/journal.pbio.3001559 (PMC8906591; doi:10.1371/journal.pbio.3001559)
Supplement: S3 Text — (PDF) [file pbio.3001559.s003.pdf]

S3 Text: Approximate dating of the timing of GRC evolution in *B. coprophila*

We used Baltic amber records to roughly estimate the date of the introgression of GRCs from Cecidomyiidae to Sciaridae. The records of the early diversification of the Sciaridae family found in the amber are dated to be ~44myo [1,2]. Supposedly, the common ancestor of the family is a bit older than that, therefore, we roughly estimate the common ancestor of Sciaridae to be 50 mya. We can use this estimate to time calibrate the phylogeny of 340 BUSCO genes (see **Fig 4**). Using this logic, the common ancestors of Sciaridae and Cecidomyiidae was 147 mya (calculated using the sum of branches from Sciaridae backwards). This agrees with both fossil evidence suggesting the base of the Sciaroidea clade (which includes both Cecidomyiidae and Sciaridae) is a minimum of 180 mya, and the dated Diptera phylogeny suggesting that the common ancestor of Cecidomyiidae and Sciaridae is 120-165 mya [3]. The isolation of Sciaridae then happened ~37 my after the split with Cecidomyiidae. We hypothesise the introgression must have happened after that as no other Bibionomorpha families have GRCs nor show any other signs of hybridization with ancestors of Cecidomyiidae [4,5]. The hybridisation therefore probably happened on the Sciaridae branch before the diversification of the family, approximately 114 - 50 mya and between 37 - 101 my after the split of the original ancestors of the two families.

These calculations must be taken with a large grain of salt, as the substitution rates change over time and our calibration is relatively crude as it uses only one reference point. However, it suggests that hybridisation of extremely divergent species (37 - 101 my) can have important evolutionary consequences. This is not the first record of viable hybrids of two extremely diverged animals. Recently a successful hybridization of Russian Sturgeon and American Paddlefish was accomplished [6]. However, this was a lab-generated hybrid,

not a result of mating in the wild. Other hybridisation events of diverged animals that resulted in gene flow are found in *Nasonia wasps* [7], sea squirt [8], and burrowing frogs [9]. The B chromosome in *Nasonia* wasps is thought to have arisen through hybridization with a *Trichomalopsis* wasp, the two lineages are estimated to have diverged 2.6 mya [10]. The B chromosome also has a substantial effect on *Nasonia* wasps, as it affects sex determination in individuals that carry it. The sea squirt gene flow appeared after secondary contact of more than 3 my of divergence between the two lineages [8], which is already an upper boundary of known systems with ongoing gene flow. Gene flow between more remote lineages, as in burrowing frogs, seems to be facilitated by polyploidisation [9]. A similar case is found in *Arabidopsis lyrata* and *A. arenosa* species complex. Both those species have diploid and tetraploid forms and while the diploid variants are fully reproductively isolated, the tetraploid variants form viable hybrids generating an indirect route for gene flow between these ~20my diverged lineages [11]. It appears that hybridization events of extremely diverged species are always associated with polyploidy, which may be more stable as recombination will not break up already-working within-subgenome co-adapted genes. It seems likely, given that the size of the GRCs in *B. coprophila* is comparable to the size of the entire Cecidomyiid genome (the genome size of *Mayetiola destructor,* for example, is 158Mb; [12]), that the originally introgressed GRCs in the common ancestor of Sciaridae carried a full genomic copy of the ancestral Cecidomyiidae genome. We speculate that GRCs originated in Sciaridae through introgression of a haploid cecidomyiid genome. The introgression might have directly resulted in GRCs or was followed by restriction of the introgressed genome to the germline. Sciaridae species therefore represent a rare case of germ-line specific polyploids.

References

1. Roschmann F, Morhig W. Die trauermucken des sächsischen bernsteins aus dem

untermiozän von Bitterfeld/Deutschland (Diptera, Sciaridae). Dtsch Entomol Zeitschrift. 1995;42: 17–54. doi:10.1002/mmnd.19950420103

2. Blagoderov V, Grimaldi D. Fossil Sciaroidea (Diptera) in Cretaceous ambers, exclusive of Cecidomyiidae, Sciaridae, and Keroplatidae. Am Museum Novit. 2004;3433: 1–76. doi:10.1206/0003-0082(2004)433<0001:fsdica>2.0.co;2

3. Wiegmann BM, Trautwein M, Winkler I, Barr N, Kim J-W, Lambkin C, et al. Episodic radiations in the fly tree of life. Proc Natl Acad Sci. 2011;108: 5690–5695. doi:10.1073/pnas.1012675108/-/DCSupplemental.www.pnas.org/cgi/doi/10.1073/pnas.1012675108

4. Le Calvez J. Morphologie et comportement des chromosomes dans la spermatogenese se quelques Mycetophilides. Chromosoma. 1947; 137–165. doi:10.1007/BF00319473

5. Fahmy OG. The mechanism of chromosome pairing during meiosis in male *Apolipthisa subincana* (Mycetophilidae, Diptera). J Genet. 1949;49: 246–263. doi:10.1007/BF02986079

6. Káldy J, Mozsár A, Fazekas G, Farkas M, Fazekas DL, Fazekas GL, et al. Hybridization of russian sturgeon (*Acipenser gueldenstaedtii*, Brandt and Ratzeberg, 1833) and american paddlefish (*Polyodon spathula*, Walbaum 1792) and evaluation of their progeny. Genes (Basel). 2020;11: 1–17. doi:10.3390/genes11070753

7. McAllister BF, Werren JH. Hybrid origin of a B chromosome (PSR) in the parasitic wasp *Nasonia vitripennis*. Chromosoma. 1997;106: 243–253. doi:10.1007/s004120050245

8. Roux C, Tsagkogeorga G, Bierne N, Galtier N. Crossing the species barrier: Genomic hotspots of introgression between two highly divergent ciona intestinalis species. Mol Biol Evol. 2013;30: 1574–1587. doi:10.1093/molbev/mst066

9. Novikova PY, Brennan IG, Booker W, Mahony M, Doughty P, Lemmon AR, et al.

Polyploidy breaks speciation barriers in Australian burrowing frogs *Neobatrachus*. PLoS Genet. 2020;16: 1–24. doi:10.1371/journal.pgen.1008769

10. Martinson EO, Mrinalini, Kelkar YD, Chang CH, Werren JH. The Evolution of Venom by Co-option of Single-Copy Genes. Curr Biol. 2017;27: 2007-2013.e8. doi:10.1016/j.cub.2017.05.032

11. Lafon-Placette C, Johannessen IM, Hornslien KS, Ali MF, Bjerkan KN, Bramsiepe J, et al. Endosperm-based hybridization barriers explain the pattern of gene flow between *Arabidopsis lyrata* and *Arabidopsis arenosa* in Central Europe. Proc Natl Acad Sci U S A. 2017;114: E1027–E1035. doi:10.1073/pnas.1615123114

12. Stuart JJ, Chen M, Harris MO. Hessian fly. Publ from USDA-ARS / UNL Fac US. 2008.
