# Supplementary material for: Gene-rich germline-restricted chromosomes in black-winged fungus gnats evolved through hybridization
Source: PLoS Biol. 2022 Feb 25;20(2):e3001559. doi: 10.1371/journal.pbio.3001559 (PMC8906591; doi:10.1371/journal.pbio.3001559)
Supplement: S1 Table — Scripts and analysis documentation describing how we generated figures/tables is available at https://github.com/RossLab/Bradysia-GRCs and at 10.5281/zenodo.5884857. All data to generate the figures within this manuscript are located at https://github.com/RossLab/Bradysia-GRCs/blob/master/tables/figure_data.tar.gz. The name of the data used to generate each figure is listed in this table. (PDF) [file pbio.3001559.s004.pdf]

S1 Table. Location of data used to generate figures in main text and supplementary figures. Scripts and analysis documentation describing how we generated figures/tables is available at https://github.com/RossLab/Bradysia-GRCs and at 10.5281/zenodo.5884857. All data to generate the figures within this manuscript are located at https://github.com/RossLab/Bradysia-GRCs/blob/master/tables/figure_data.tar.gz. The name of the data used to generate each figure is listed in this table.

Figure	Description	Data Type	Data Name
Fig 1C	histogram of log2 germline/soma scaffold coverage	table	table.covdiff.germ.soma.tsv
Fig 1D	k-mer 2D histogram comparing germline and somatic libraries	table	headlib_vs_testeslib_kmer-main.mx
Fig 2A/B	A-pie chart of GRC gene categories; B-bar plot of pairwise homologs in genome	table	homologs_Bcop_genome.tsv
Fig 3A	Histogram of GRC scaffold coverage	table	GRC_scaffolds_chrom_assignment.tsv
Fig 3B	Comparison of scaffold coverage of GRC-GRC homologs	table	GRC_GRC_homolog_cov.tsv
Fig 3C	E.g. of mean gene coverage of homologs along a GRC1-GRC2 collinear block	table	fig3C.tsv
Fig 4B	Bar plot, and boxplots showing GRC BUSCO gene phylogenetic placement, bootstrap, and branch length	table	BUSCO_GRC_phylogeny_table.tsv
Fig 4C	Bar plot, and boxplots showing core BUSCO gene phylogenetic placement, bootstrap, and branch length	table	BUSCO_core_phylogeny_table.tsv

Fig 4D	Concatenated phylogeny of GRC-core BUSCOs where GRC gene is in Cecidomyiidae	gene alignments	fig5D/
Fig 4E	Concatenated phylogeny of GRC-core BUSCOs where GRC gene is in Sciaridae	gene alignments	fig5E/
Supplementary Fig 1	Unfiltered geneme assembly blobplot	table	Bcop.blobDB.bestsumorder.table.txt
Supplementary Fig 2A/B	K-mer score histogram for Illumina genome assembly and Pacbio assembly	table	scaffold_assignment_tab_full.tsv
Supplementary Fig 3A-F	Historgrams for amino acid identity for homolog pairs in genome, separated by homolog type	table	homologs_Bcop_genome.tsv
Supplementary Fig 4	GRC-assignment plot showing normal curves	table	GRC_scaffolds_assignment_tab.tsv
Supplementary Fig 5A	Histogram of all block assignments	table	GRC-GRC_collinear_assignment_tab.tsv
Supplementary Fig 5B	E.g. of a GRC1-GRC2 block	table	Suppfig5b.tsv
Supplementary Fig 5C	E.g. of a GRC1-GRC1 block	table	Suppfig5c.tsv
Supplementary Fig 5D	E.g of a GRC2-GRC2 block	table	Suppfig5d.tsv
Supplementary Fig 6	BUSCO results for all Sciaroidea species	table	BUSCO_summary_Sciaroidea.tsv
Supplementary Fig 7A-K	Bar plot, and boxplots showing BUSCO gene phylogenetic placement, bootstrap, and branch length separated by BUSCO category	table	BUSCO_GRC_phylogeny_table.tsv; BUSCO_core_phylogeny_table.tsv

Supplementary Fig 8	Heatmap of AA composition bias in BUSCO genes	table	BUSCO_aa_composition.tsv
Supplementary Fig 9A-F	E.g of BUSCO phylogenies with different topologies	gene alignments	SFig9/
Supplementary Fig 10	Plot of branch lengths of GRC gene copies falling within Cecidomyiidae vs. Sciaridae	table	BUSCO_GRC_phylogeny_table.tsv
Supplementary Fig 11A-C	Plots comparing identity of homologs that have a BLAST hit in both core B. coprophila genome and M. destructor genome	table	Mdes_vs_Bcopcore_GRC_identity_comp.tsv
Supplementary Fig 11D	Barplot summarising how many genes are in Supplementary Fig 11A and why some genes were filtered out	table	Mdes_vs_GRC_homolog.tsv; homologs_bcop.tsv
