# Supplementary material for: Gene-rich germline-restricted chromosomes in black-winged fungus gnats evolved through hybridization
Source: PLoS Biol. 2022 Feb 25;20(2):e3001559. doi: 10.1371/journal.pbio.3001559 (PMC8906591; doi:10.1371/journal.pbio.3001559)
Supplement: S2 Table — The short-read assembly was used for gene prediction as it was closer to the expected genome size compared to the long-read assembly and also was more complete according to BUSCO assessment. (PDF) [file pbio.3001559.s005.pdf]

S2 Table. Summary statistics for the short read and long read assemblies used in this study. The short read assembly was used for gene prediction as it was closer to the expected genome size compared to the long read assembly and also was more complete according to BUSCO assessment.

	Short read (Illumina)	Long read (PacBio)
Size	398 MB	415 Mb
# scaffolds	46,532	3505
N50	18.9 Kb	576 Kb
L50	5203	135
GC	35.4%	35.8%
BUSCO completeness	98.3%	93.6%
