# Supplementary material for: Gene-rich germline-restricted chromosomes in black-winged fungus gnats evolved through hybridization
Source: PLoS Biol. 2022 Feb 25;20(2):e3001559. doi: 10.1371/journal.pbio.3001559 (PMC8906591; doi:10.1371/journal.pbio.3001559)
Supplement: S3 Table — Chromosome assignment using the k-mer method is shown in the first column and the assignment with the coverage method is shown in the second column with the total size of unclassified sequence belonging to that category shown in the third column. In the k-mer assignment column, a “c” indicates cases where the majority k-mers from one chromosome mapped to that scaffold but the k-mer identification score (see Fig 2A) was too low to support a confident assignment with this method. (PDF) [file pbio.3001559.s006.pdf]

**S3 Table. Size of unclassified scaffolds from k-mer and coverage identification methods.** Chromosome assignment using the k-mer method is shown in the first column and the assignment with the coverage method is shown in the second column with the total size of unclassified sequence belonging to that category shown in the third column. In the k-mer assignment column, a "c" indicates cases where the majority k-mers from one chromosome mapped to that scaffold but the k-mer identification score (see **Supplementary Fig. 2A**) was too low to support a confident assignment with this method.

| K-mer assignment | Coverage assignment | Size (Mb) |
|---|---|---|
| X | A | 3.3 |
| X | GRC | 0.1 |
| Xc | X | 2.4 |
| A | X | 0.4 |
| A | GRC | 0.1 |
| Ac | A | 9 |
| GRC | X | 0.7 |
| GRC | A | 5.4 |
| GRCc | GRC | 6.8 |
