# Supplementary material for: Gene-rich germline-restricted chromosomes in black-winged fungus gnats evolved through hybridization
Source: PLoS Biol. 2022 Feb 25;20(2):e3001559. doi: 10.1371/journal.pbio.3001559 (PMC8906591; doi:10.1371/journal.pbio.3001559)
Supplement: S4 Table — (PDF) [file pbio.3001559.s007.pdf]

S4 Table. Size and proportion of our genome assembly anchored to the *B. coprophila* reference genome [43] and the number of GRC homologs and number of GRC collinear blocks anchored to each chromosome in the reference assembly.

	A-II	A-III	A-IV	X
Size (Mb)	48-62	66-71	88-94	48-62
Proportion anchored	20-46%	8-19%	37-52%	93-100%
GRC homologs (number genes)	128	7	108	119
GRC collinear blocks	14	1	12	12

Reference

43. Urban JM, Foulk MS, Bliss JE, Coleman CM, Lu N, Mazloom R, et al. High contiguity de novo genome assembly and DNA modication analyses for the fungus fly *Sciara coprophila*, using single molecule sequencing. BMC Genomics. 2021;22: 1–23. doi:10.1186/s12864-021-07926-2
