# Supplementary material for: Gene-rich germline-restricted chromosomes in black-winged fungus gnats evolved through hybridization
Source: PLoS Biol. 2022 Feb 25;20(2):e3001559. doi: 10.1371/journal.pbio.3001559 (PMC8906591; doi:10.1371/journal.pbio.3001559)
Supplement: S5 Table — BUSCO assessment was conducted with the insecta_odb10 database. The genomic location of genes was identified with both coverage and k-mer identification techniques. Individual gene trees were examined for all categories of BUSCOs listed below (Fig 4B and 4C, S7 Fig) and concatenated phylogenies were generated for categories indicated with * (Fig 4D and 4E). BUSCO categories including unassigned genes (NA assignment) are excluded from this table. (PDF) [file pbio.3001559.s008.pdf]

S5 Table. Genomic location of universal single-copy orthologs (BUSCO) in *Bradysia coprophila*. BUSCO assessment was conducted with the insecta_odb10 database. The genomic location of genes was identified with both coverage and k-mer identification techniques. Individual gene trees were examined for all categories of BUSCOs listed below (**Fig 4B/C; S7 Fig)** and concatenated phylogenies were generated for categories indicated with * (**Fig 4D/E)**. BUSCO categories including unassigned genes (NA assignment) are excluded from this table.

BUSCO type	Chromosome	Frequency	GRC related
Single-copy	A	521	No
	X	182	No
	GRC	106	Yes
Duplicated	A-GRC*	291	Yes
	X-GRC*	81	Yes
	GRC-GRC	18	Yes
	A-A	6	No
	A-X	1	No
Multi-copy	A-GRC-GRC	56	Yes
	X-GRC-GRC	30	Yes
	GRC-GRC-GRC	3	Yes
	A-A-GRC	3	Yes
	A-X-GRC	2	Yes
	A-GRC-GRC-GRC	1	Yes
	X-X-GRC-GRC-GRC	1	Yes
