# Supplementary material for: Gene-rich germline-restricted chromosomes in black-winged fungus gnats evolved through hybridization
Source: PLoS Biol. 2022 Feb 25;20(2):e3001559. doi: 10.1371/journal.pbio.3001559 (PMC8906591; doi:10.1371/journal.pbio.3001559)
Supplement: S6 Table — Only GRC genes that fall within the Cecidomyiidae or Sciaridae clade are shown and only genes that have a scaffold coverage ranging from 18× to 35×. More genes on GRC1 fall within the Cecidomyiidae clade compared to genes on GRC2 (Fisher exact test: odds ratio = 5.25, 95% CI = 3.16 to 9.02, p < 0.0001). However, both GRC chromosomes contain some genes that are within the Cecidomyiidae clade in phylogenies. Numbers in parenthesis indicate genes that are likely present on that chromosome, but which we were unable to unambiguously classify to a specific GRC (i.e., genes with a c classification). GRC, germline-restricted chromosome. (PDF) [file pbio.3001559.s009.pdf]

S6 Table. Phylogenetic placement of GRC BUSCO genes separated by whether the genes are on GRC1 or GRC2. Only GRC genes that fall within the Cecidomyiidae or Sciaridae clade are shown and only genes that have a scaffold coverage ranging from 18x-35x. More genes on GRC1 fall within the Cecidomyiidae clade compared to genes on GRC2 (Fisher's exact test: odds ratio: 5.25, CI: 3.16-9.02, p<0.0001). However, both GRC chromosomes contain some genes that are within the Cecidomyiidae clade in phylogenies. Numbers in parenthesis indicate genes that are likely present on that chromosome, but which we were unable to unambiguously classify to a specific GRC (i.e. genes with a c classification).

	Phylogenetic placement	
	Cecidomyiidae	Sciaridae
GRC1	205 (6)	24 (1)
GRC2	157 (13)	97 (2)
