# Supplementary material for: Gene-rich germline-restricted chromosomes in black-winged fungus gnats evolved through hybridization
Source: PLoS Biol. 2022 Feb 25;20(2):e3001559. doi: 10.1371/journal.pbio.3001559 (PMC8906591; doi:10.1371/journal.pbio.3001559)
Supplement: S1 Fig — Reads mapping to scaffolds with a GC content between 0.14 and 0.51 and a coverage higher than 7 were retained for the final assembly. Location of data used to generate this figure is specified in S1 Table. (PDF) [file pbio.3001559.s010.pdf]

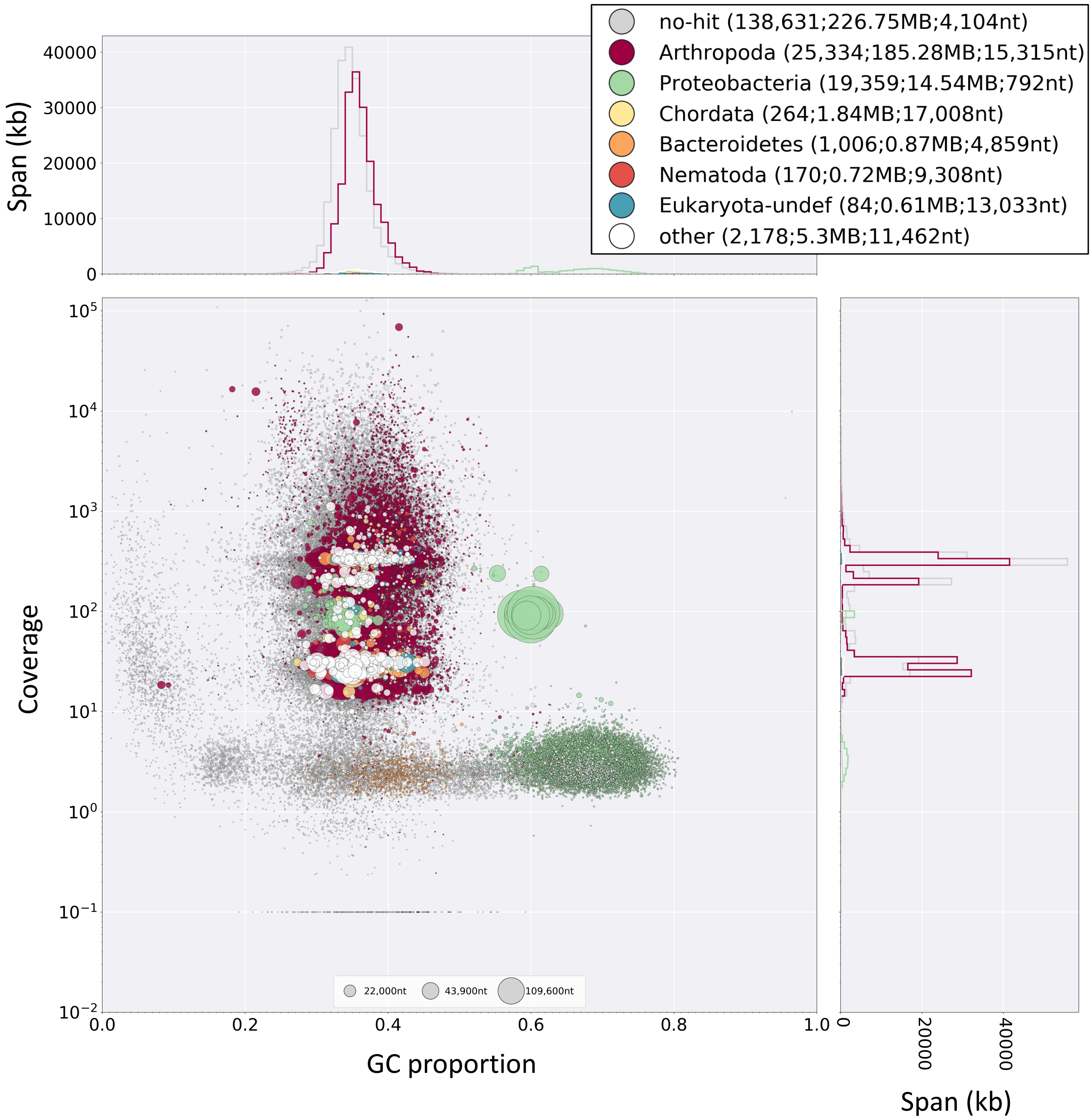

S1 Fig. Blobplot of unfiltered assembly generated from both germ and somatic libraries showing scaffold coverage vs. scaffold GC (size of dot indicates scaffold size and colour taxonomic assignment). Reads mapping to scaffolds with a GC content between 0.14 and 0.51 and a coverage higher than 7 were retained for the final assembly. Location of data used to generate this figure is specified in **S1 Table.**
