# Supplementary material for: Gene-rich germline-restricted chromosomes in black-winged fungus gnats evolved through hybridization
Source: PLoS Biol. 2022 Feb 25;20(2):e3001559. doi: 10.1371/journal.pbio.3001559 (PMC8906591; doi:10.1371/journal.pbio.3001559)
Supplement: S2 Fig — (A) Histogram of k-mer assignment scores for scaffolds of each chromosome type in the short-read assembly used throughout the manuscript. The score is defined as the number of k-mers with an exact match to the scaffold from the chromosome type with the majority of k-mers matching the scaffold, divided by the scaffold length. For GRC scaffolds (orange), we only assigned scaffolds with a score higher than 0.8 as GRC scaffolds, while for autosomal and X chromosome scaffolds (green and blue, respectively) we assigned scaffolds with a score higher than 0.4, as the GRC scaffolds had a more distinct k-mer profile (i.e., higher k-mer score) than autosomes and the X chromosome. (B) Histogram of k-mer assignment scores in the long-read assembly (see S2 Text). The scores are substantially lower, especially for differentiating autosomes and the X chromosome. This assembly was used only for anchoring GRC genes in longer blocks for the collinearity analysis. Location of data used to generate this figure is specified in S1 Table. GRC, germline-restricted chromosome. (PDF) [file pbio.3001559.s011.pdf]

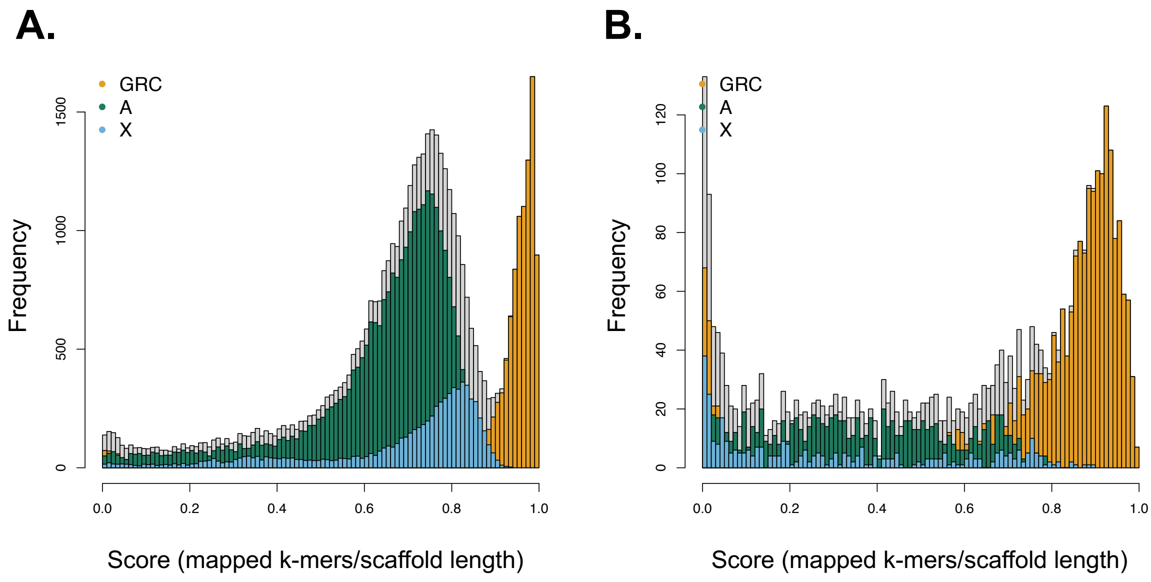

S2 Fig. Distributions of scores used in the k-mer identification technique. A. Histogram of k-mer assignment scores for scaffolds of each chromosome type in the short-read assembly used throughout the manuscript. The score is defined as the number of k-mers with an exact match to the scaffold from the chromosome type with the majority of k-mers matching the scaffold, divided by the scaffold length. For GRC scaffolds (orange), we only assigned scaffolds with a score higher than 0.8 as GRC scaffolds, while for autosomal and X chromosome scaffolds (green and blue respectively) we assigned scaffolds with a score higher than 0.4, as the GRC scaffolds had a more distinct k-mer profile (i.e. higher k-mer score) than autosomes and the X chromosome. **B.** Histogram of k-mer assignment scores in the long read assembly (See **S2 Text**). The scores are substantially lower, especially for differentiating autosomes and the X chromosome. This assembly was used only for anchoring GRC genes in longer blocks for the collinearity analysis. Location of data used to generate this figure is specified in **S1 Table.**
