# Supplementary material for: Gene-rich germline-restricted chromosomes in black-winged fungus gnats evolved through hybridization
Source: PLoS Biol. 2022 Feb 25;20(2):e3001559. doi: 10.1371/journal.pbio.3001559 (PMC8906591; doi:10.1371/journal.pbio.3001559)
Supplement: S3 Fig — Plots are separated by the location of genes involved in the blast hit, with (A) showing autosomal-autosomal homologs, (B) showing GRC-autosomal homologs, (C) showing GRC-GRC homologs, (D) showing GRC-X chromosome homologs, (E) showing autosomal-X chromosome homologs, and (F) showing X chromosome-X chromosome homologs. We set a threshold of 40% identity and genes covering at least 60% of each other to assign reciprocal blast hits, so we would to capture only hits that spanned most of the length of each gene. Location of data used to generate this figure is specified in S1 Table. GRC, germline-restricted chromosome. (PDF) [file pbio.3001559.s012.pdf]

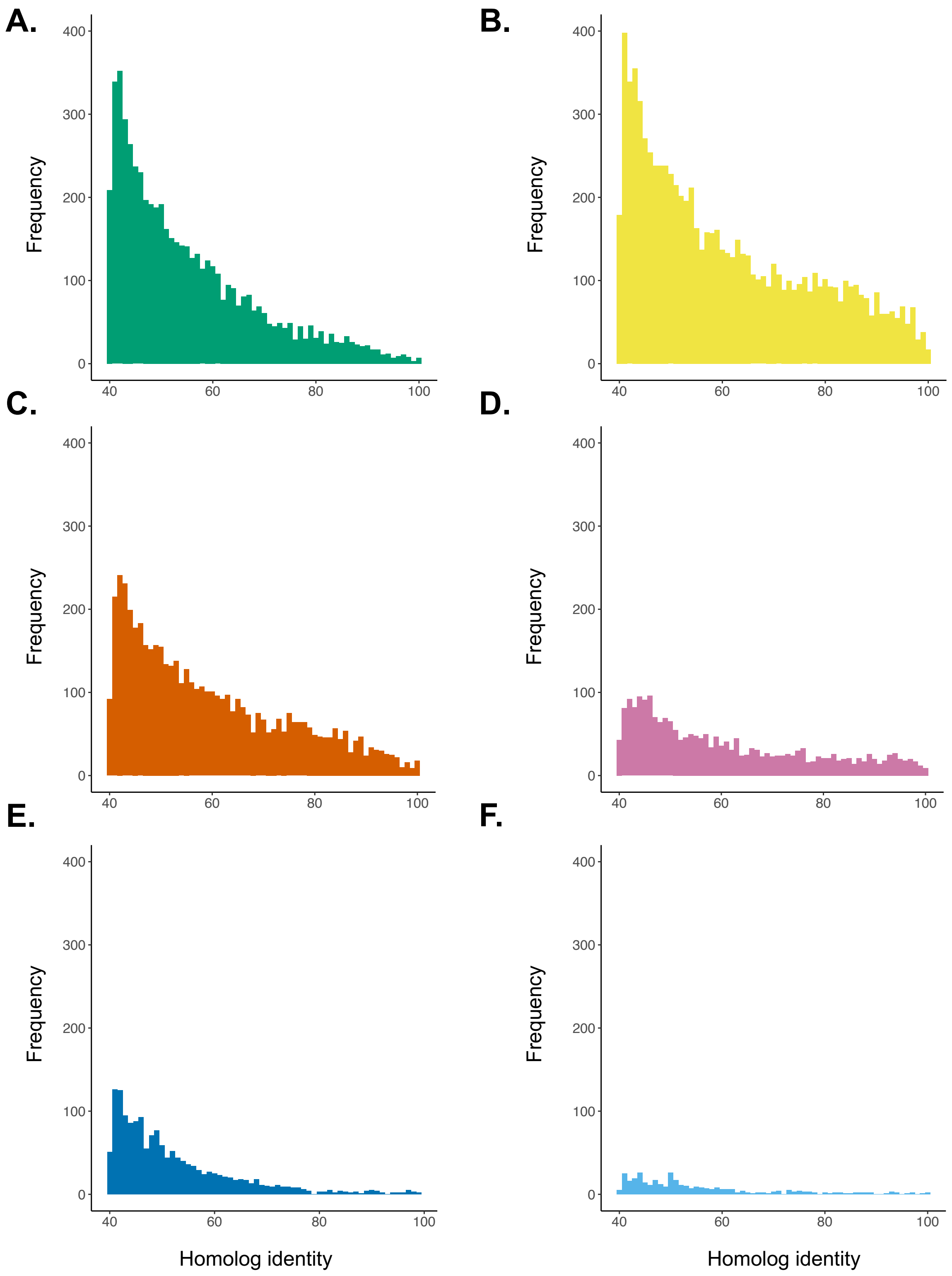

S3 Fig. Amino acid identity for reciprocal blast hits within the *B. coprophila* genome. Plots are separated by the location of genes involved in the blast hit, with **A.** showing autosomal-autosomal homologs, **B.** showing GRC-autosomal homologs, **C.** showing GRC-

GRC homologs, **D.** showing GRC-X chromosome homologs, **E** showing autosomal-X chromosome homologs and **F.** showing X chromosome-X chromosome homologs. We set a threshold of 40% identity and genes covering at least 60% of each other to assign reciprocal blast hits, so we would to capture only hits that spanned most of the length of each gene. Location of data used to generate this figure is specified in **S1 Table.**
