# Supplementary material for: Gene-rich germline-restricted chromosomes in black-winged fungus gnats evolved through hybridization
Source: PLoS Biol. 2022 Feb 25;20(2):e3001559. doi: 10.1371/journal.pbio.3001559 (PMC8906591; doi:10.1371/journal.pbio.3001559)
Supplement: S4 Fig — Scaffolds longer than 5,000 bp, between 18× and 35× coverage and assigned to the GRCs were used to generate a density plot from which 2 normal curves were drawn (red curve = GRC1, blue curve = GRC2, dashed lines = means). We assigned each scaffold to GRC1 or GRC2 by taking the scaffold coverage and determining whether it was more likely to belong to GRC1 or GRC2. Location of data used to generate this figure is specified in S1 Table. GRC, germline-restricted chromosome. (PDF) [file pbio.3001559.s013.pdf]

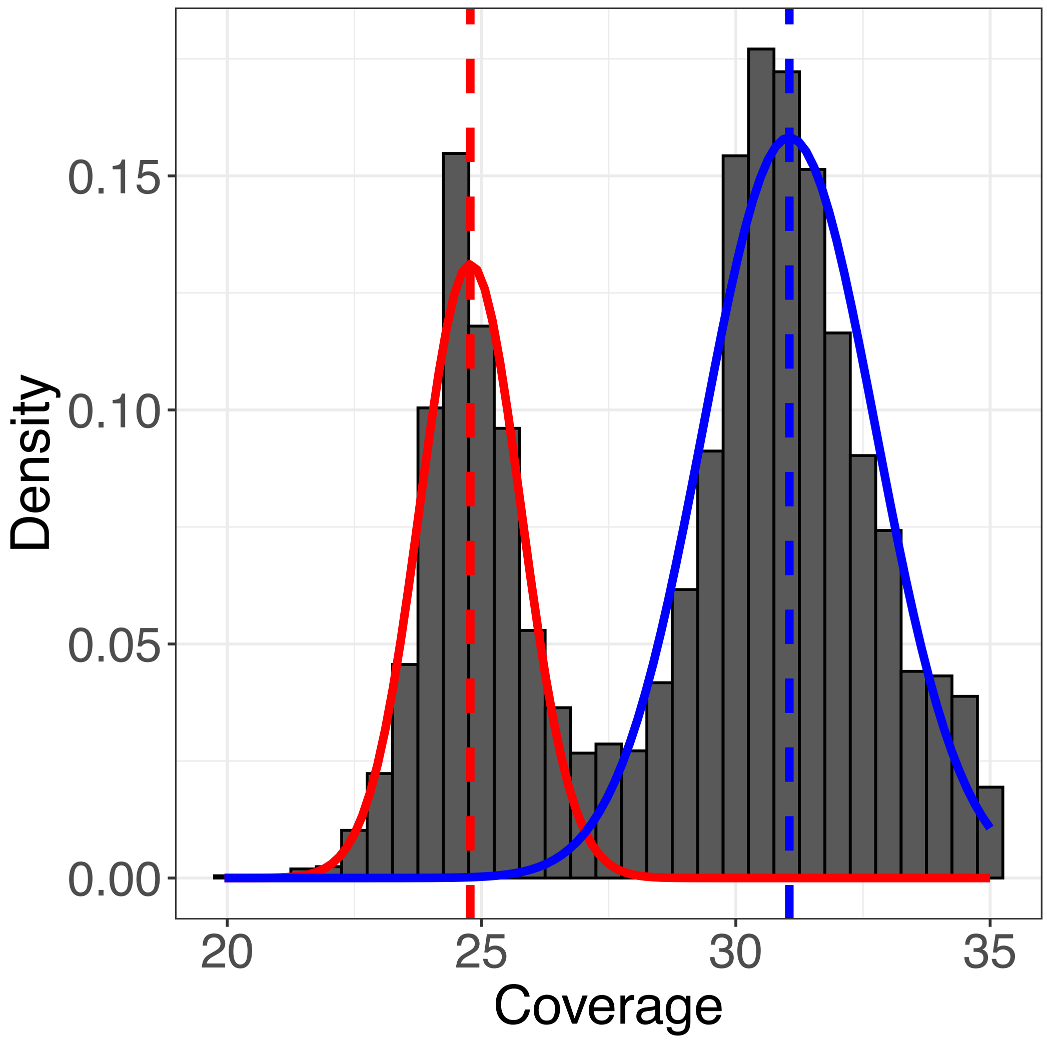

S4 Fig. Assignment of scaffolds to GRC1 or GRC2. Scaffolds longer than 5,000 bp, between 18x and 35x coverage and assigned to the GRCs were used to generate a density plot from which two normal curves were drawn (red curve= GRC1, blue curve=GRC2, dashed lines= means). We assigned each scaffold to GRC1 or GRC2 by taking the scaffold coverage and determining whether it was more likely to belong to GRC1 or GRC2. Location of data used to generate this figure is specified in **S1 Table.**
