# Supplementary material for: Gene-rich germline-restricted chromosomes in black-winged fungus gnats evolved through hybridization
Source: PLoS Biol. 2022 Feb 25;20(2):e3001559. doi: 10.1371/journal.pbio.3001559 (PMC8906591; doi:10.1371/journal.pbio.3001559)
Supplement: S5 Fig — (A) Barplot showing the number of the GRC-GRC collinear blocks we were able to assign to GRC1/GRC2. Blocks in which all but one gene in the same block were assigned to the same GRC are denoted with a c (i.e., GRC1-GRC2c). We were unable to assign several blocks either because one block did not have a consistent GRC assignment or because one block did not have enough genes on it which were assigned to one of the GRCs (because the scaffold lengths for the genes in these blocks were less than 5,000 bp). (B–D) Examples of gene coverages along a GRC1-GRC2 collinear block (B), a GRC1-GRC1 collinear block (C) and a GRC2-GRC2 collinear block (D). Location of data used to generate this figure is specified in S1 Table. GRC, germline-restricted chromosome. (PDF) [file pbio.3001559.s014.pdf]

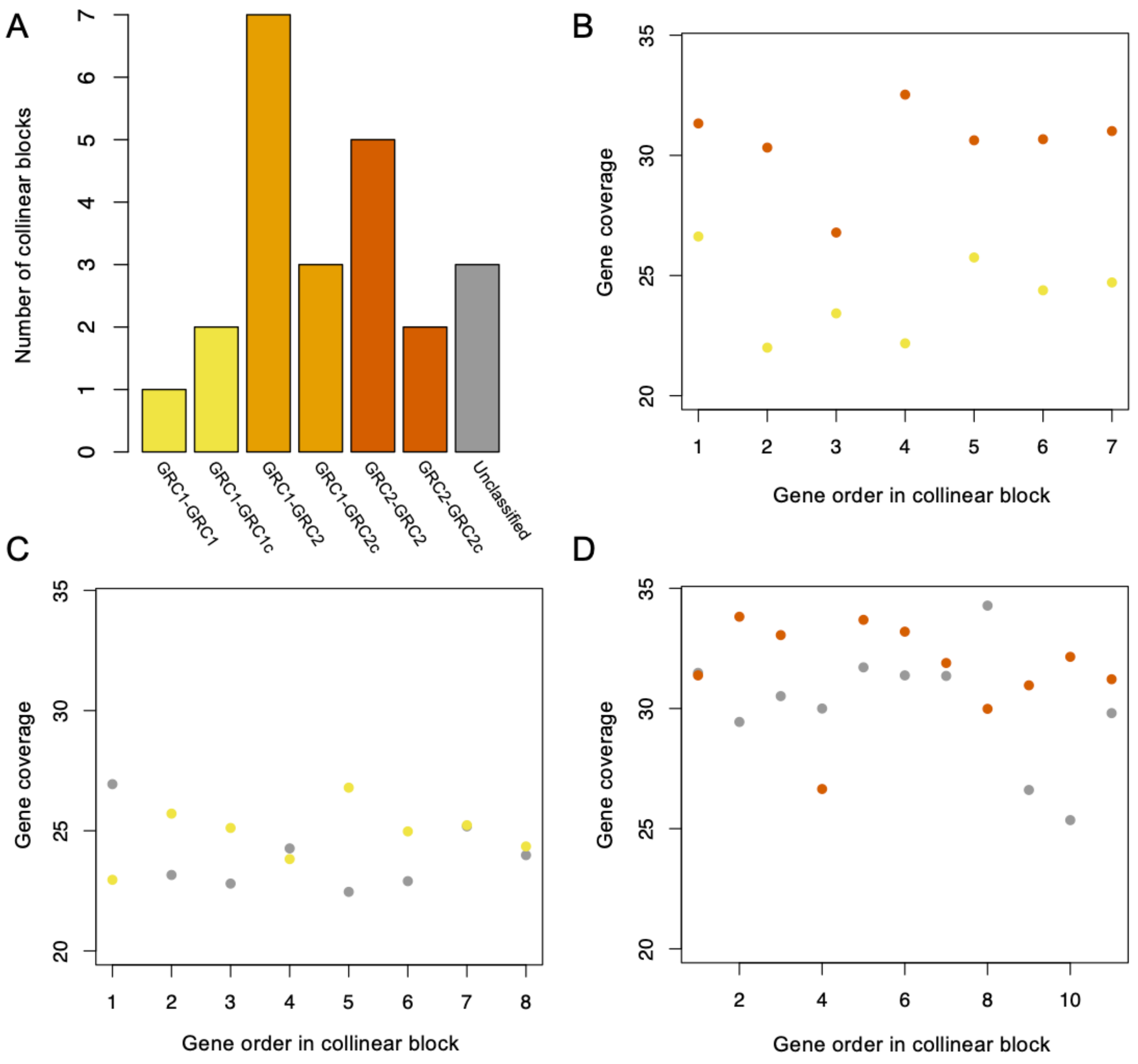

S5 Fig. Assignment of GRC-GRC collinear blocks to GRC1 or GRC2. A. barplot showing the number of the GRC-GRC collinear blocks we were able to assign to GRC1 / GRC2. Blocks in which all but one gene in the same block were assigned to the same GRC are denoted with a c (i.e. GRC1-GRC2c). We were unable to assign several blocks either because one block did not have a consistent GRC assignment or because one block did not have enough genes on it which were assigned to one of the GRCs (because the scaffold lengths for the genes in these blocks were less than 5,000bp). **B-D.** examples of gene coverages along a GRC1-GRC2 collinear block (B), a GRC1-GRC1 collinear block (C) and a

GRC2-GRC2 collinear block (D). Location of data used to generate this figure is specified in **S1 Table.**
