# Supplementary material for: Gene-rich germline-restricted chromosomes in black-winged fungus gnats evolved through hybridization
Source: PLoS Biol. 2022 Feb 25;20(2):e3001559. doi: 10.1371/journal.pbio.3001559 (PMC8906591; doi:10.1371/journal.pbio.3001559)
Supplement: S6 Fig — Exechia fusca was excluded from analyses as the proportion of complete BUSCOs was low (54%). In B. coprophila, 39.2% of the insect BUSCO genes were duplicated. Location of data used to generate this figure is specified in S1 Table. (PDF) [file pbio.3001559.s015.pdf]

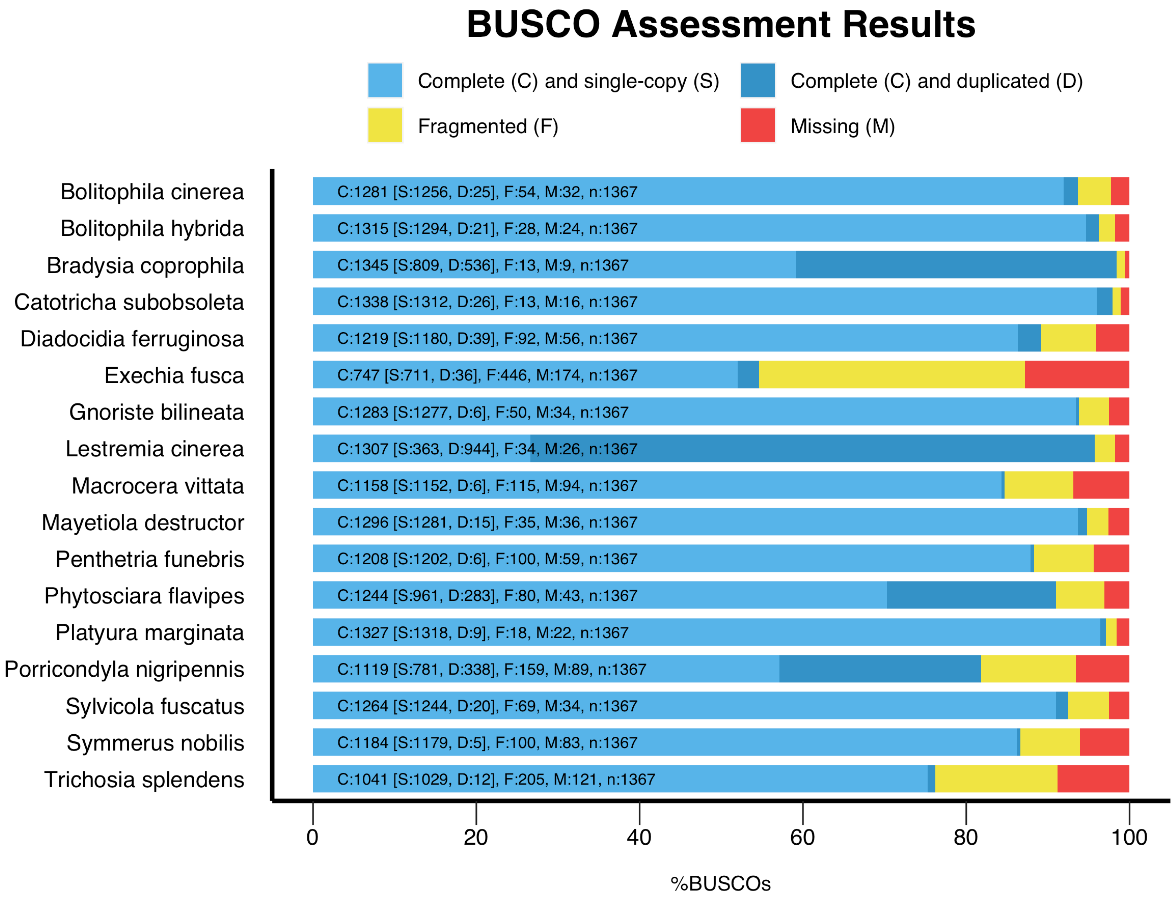

S6 Fig. Summary of universal single-copy orthologs (BUSCO) results for all Dipteran species in phylogenetic analyses. *Exechia fusca* was excluded from analyses as the proportion of complete BUSCOs was low (54%). In *Bradysia coprophila,* 39.2% of the insect BUSCO genes were duplicated. Location of data used to generate this figure is specified in **S1 Table.**
