# Supplementary material for: Gene-rich germline-restricted chromosomes in black-winged fungus gnats evolved through hybridization
Source: PLoS Biol. 2022 Feb 25;20(2):e3001559. doi: 10.1371/journal.pbio.3001559 (PMC8906591; doi:10.1371/journal.pbio.3001559)
Supplement: S7 Fig — For each BUSCO category, the barplot summarizes how many genes fall within the Sciaridae clade (teal), Cecidomyiidae clade (purple), or other (gray), the upper right boxplot shows the bootstrap values of the closest node for each gene within that category, and the bottom right boxplot shows the branch lengths for each gene within that category. Some categories are shown twice if they contain both GRC and core genes as these gene types were plotted separately. Fig 4B and 4C shows a summary of all GRC BUSCO genes and all core genome BUSCO genes, respectively. Location of data used to generate this figure is specified in S1 Table. GRC, germline-restricted chromosome. (PDF) [file pbio.3001559.s016.pdf]

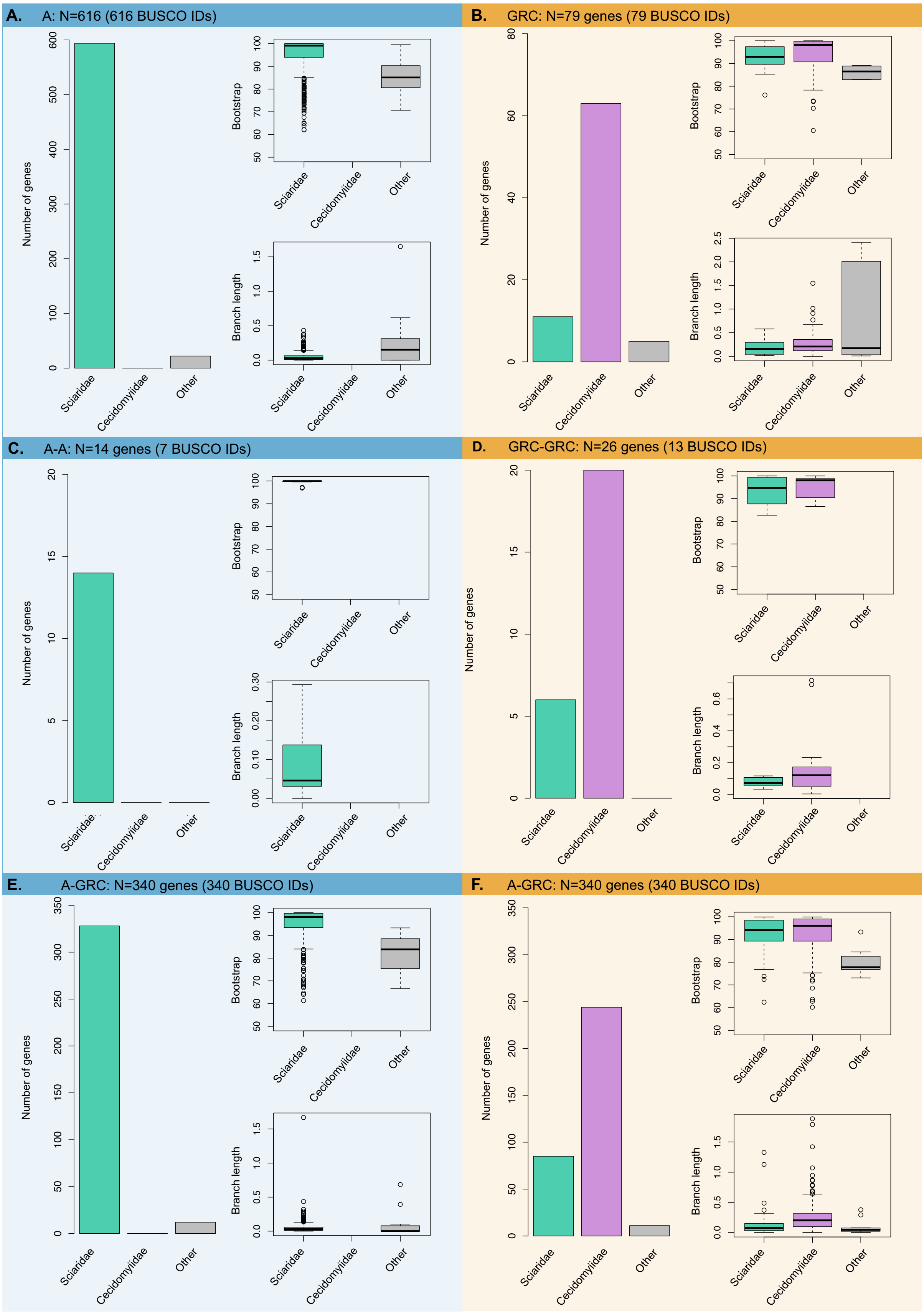

A.
A: N=616 (616 BUSCO IDs)
Number of genes
Bootstrap
Branch length
Sciaridae
Cecidomyiidae
Other
B.
GRC: N=79 genes (79 BUSCO IDs)
Number of genes
Bootstrap
Branch length
Sciaridae
Cecidomyiidae
Other
C.
A-A: N=14 genes (7 BUSCO IDs)
Number of genes
Bootstrap
Branch length
Sciaridae
Cecidomyiidae
Other
D.
GRC-GRC: N=26 genes (13 BUSCO IDs)
Number of genes
Bootstrap
Branch length
Sciaridae
Cecidomyiidae
Other
E.
A-GRC: N=340 genes (340 BUSCO IDs)
Number of genes
Bootstrap
Branch length
Sciaridae
Cecidomyiidae
Other
F.
A-GRC: N=340 genes (340 BUSCO IDs)
Number of genes
Bootstrap
Branch length
Sciaridae
Cecidomyiidae
Other

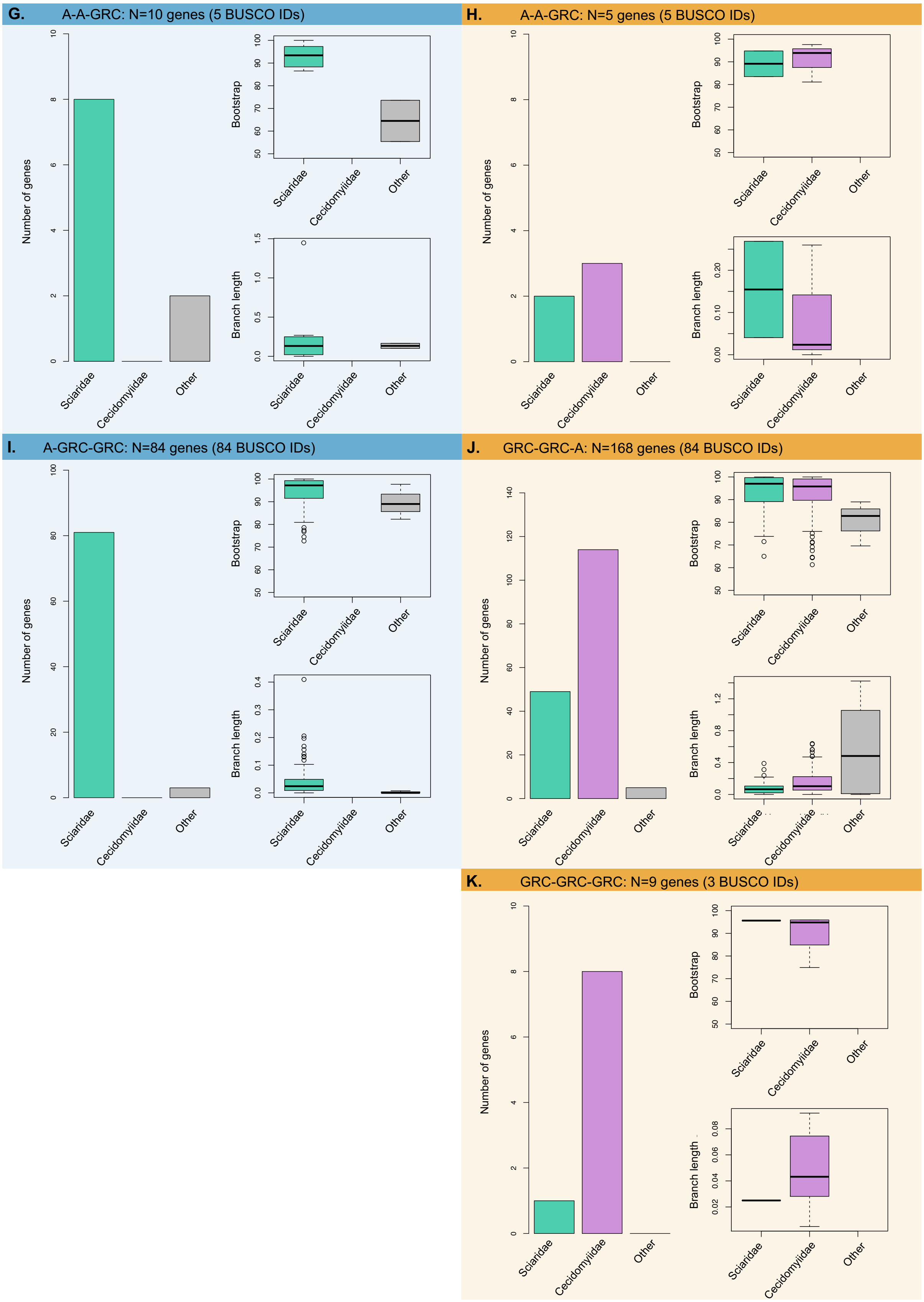

S7 Fig. plots summarising the phylogenetic position (i.e. Cecidomyiidae clade, Sciaridae clade, or other) of GRC (orange background) or core genome A genes (blue

background- autosomal or X chromosome) for all categories of BUSCO IDs separately. For each BUSCO category, the barplot summarises how many genes fall within the Sciaridae clade (teal), Cecidomyiidae clade (purple), or other (grey), the upper right boxplot shows the bootstrap values of the closest node for each gene within that category, and the bottom right boxplot shows the branch lengths for each gene within that category. Some categories are shown twice if they contain both GRC and core genes as these gene types were plotted separately. **Fig 4B/C** shows a summary of all GRC BUSCO genes and all core genome BUSCO genes respectively. Location of data used to generate this figure is specified in **S1 Table.**
