# Supplementary material for: Gene-rich germline-restricted chromosomes in black-winged fungus gnats evolved through hybridization
Source: PLoS Biol. 2022 Feb 25;20(2):e3001559. doi: 10.1371/journal.pbio.3001559 (PMC8906591; doi:10.1371/journal.pbio.3001559)
Supplement: S8 Fig — Amino acid composition analysis did not show any potential bias that could cause long-branch attraction. For the genes used for the phylogenetic analysis we calculated the amino acid composition and used a heatmap to visualize the relative frequencies of individual amino acids. Overall, there is not much variation among the analyzed species. Furthermore, the composition of GRC genes (L-Sciara_coprophilla), clustered together with other Sciaridae genomes. The only Sciara genes with deviated aa compositions were those we were unable to classify (NA-Sciara_coprophila), which might be related to the same problems we experienced when we attempted to assign chromosomes (difficult structure). Location of data used to generate this figure is specified in S1 Table. Note: Sciara_coprophila is a synonym for B. coprophila. GRC, germline-restricted chromosome. (PDF) [file pbio.3001559.s017.pdf]

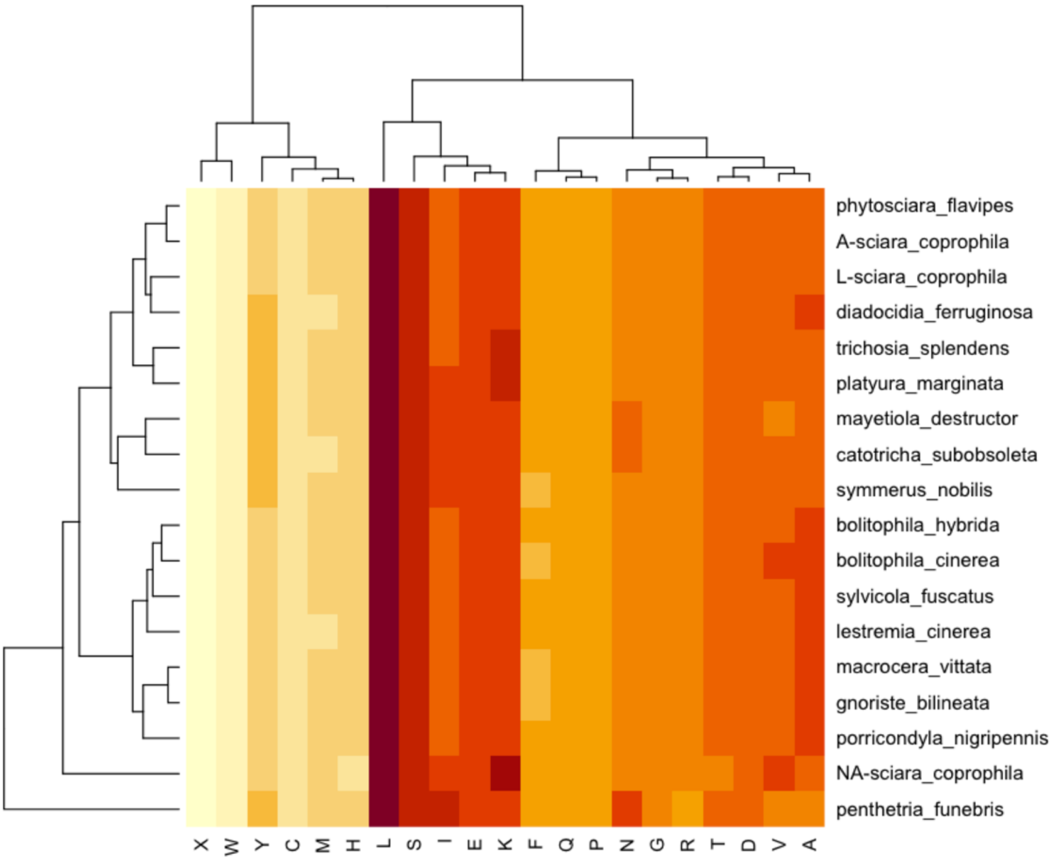

S8 Fig. Heat map of amino acid composition of BUSCO genes used in phylogenetic analyses. Amino acid composition analysis did not show any potential bias that could cause long-branch attraction. For the genes used for the phylogenetic analysis we calculated the amino acid composition and used a heat map to visualize the relative frequencies of individual amino acids. Overall, there is not much variation among the analysed species. Furthermore, the composition of GRC genes (L-*Sciara_coprophilla*), clustered together with other Sciaridae genomes. The only *Sciara* genes with deviated aa compositions were those we were unable to classify (NA-*Sciara_coprophila*), which might be related to the same problems we experienced when we attempted to assign chromosomes (difficult structure). Location of data used to generate this figure is specified in **S1 Table.** Note: *Sciara_coprophila* is a synonym for *Bradysia coprophila.*
