# Supplementary material for: Gene-rich germline-restricted chromosomes in black-winged fungus gnats evolved through hybridization
Source: PLoS Biol. 2022 Feb 25;20(2):e3001559. doi: 10.1371/journal.pbio.3001559 (PMC8906591; doi:10.1371/journal.pbio.3001559)
Supplement: S9 Fig — (A) With 1 GRC copy rooted in Cecidomyiidae, (B) with 1 GRC copy rooted in Sciaridae, (C) with 2 GRC copies both in Cecidomyiidae, (E) with 2 GRC copies, 1 in Cecidomyiidae and the other in Sciaridae, (D) with 2 GRC copies both in Sciaridae. (F) GRCs unplaced (without significant nodes) or branching with a species from any other family. Location of data used to generate this figure is specified in S1 Table. GRC, germline-restricted chromosome. (PDF) [file pbio.3001559.s018.pdf]

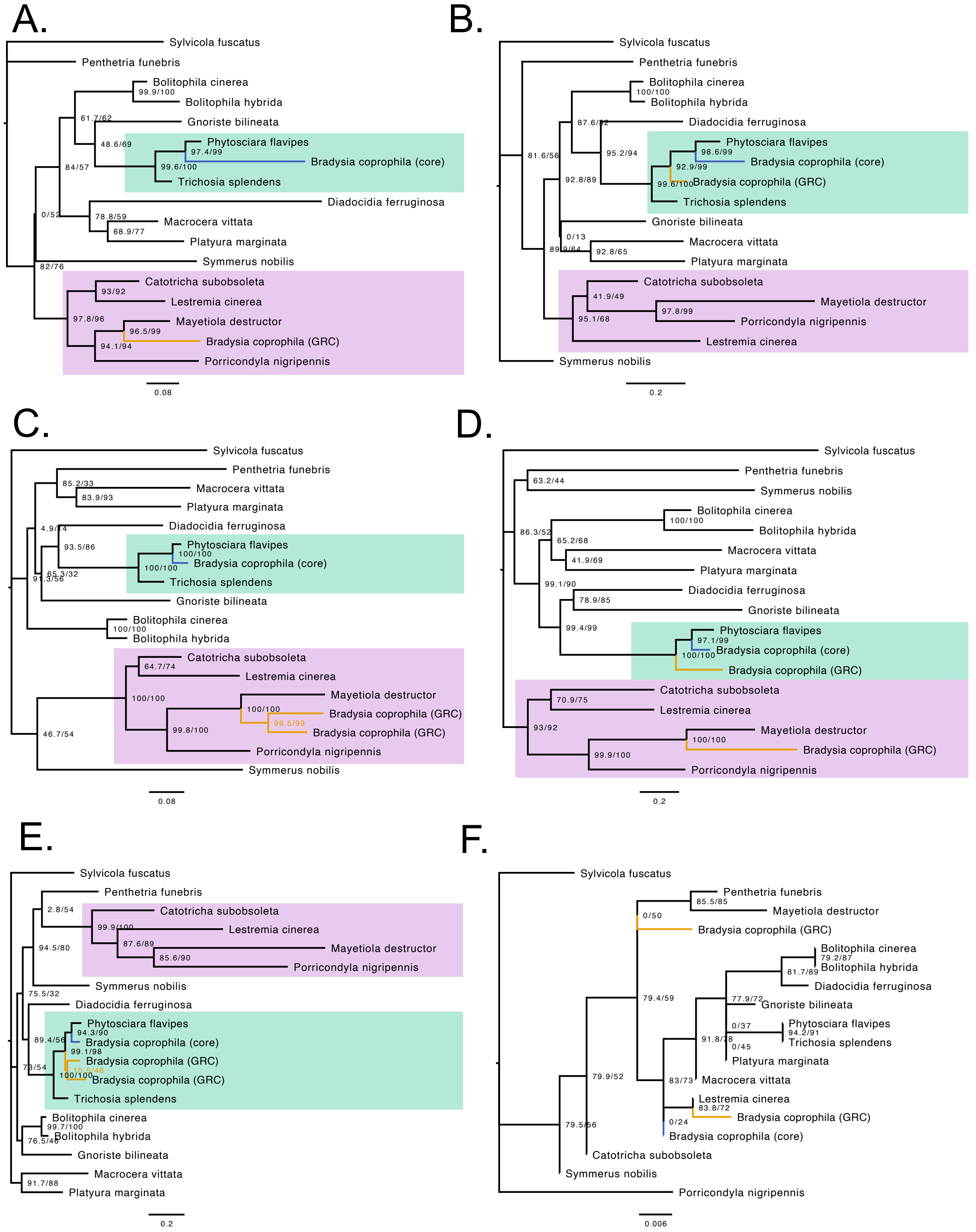

S9 Fig. Examples of GRC gene trees with various topologies. **A.** with one GRC copy rooted in Cecidomyiidae **B.** with one GRC copy rooted in Sciaridae, **C,** with two GRC copies both in Cecidomyiidae, **E**. with two GRC copies, one in Cecidomyiidae and the other in

Sciaridae, **D**. with two GRC copies both in Sciaridae. **F**. GRCs unplaced (without significant nodes) or branching with a species from any other family. Location of data used to generate this figure is specified in **S1 Table.**
