# Supplementary material for: Gene-rich germline-restricted chromosomes in black-winged fungus gnats evolved through hybridization
Source: PLoS Biol. 2022 Feb 25;20(2):e3001559. doi: 10.1371/journal.pbio.3001559 (PMC8906591; doi:10.1371/journal.pbio.3001559)
Supplement: S10 Fig — Branch length distribution of GRC copies of BUSCO genes plotted with respect to the phylogenetic position (at family level), means shown by dashed lines. Branch lengths of BUSCO genes on GRCs within Cecidomyiidae (violet) are significantly longer than branches found within Sciaridae (teal; p-value < 0.0001) suggesting the genes on GRCs found within Sciaridae might be due to gene duplications and translocations within Sciaridae after the GRCs were acquired. Location of data used to generate figure is specified in S1 Table. GRC, germline-restricted chromosome. (PDF) [file pbio.3001559.s019.pdf]

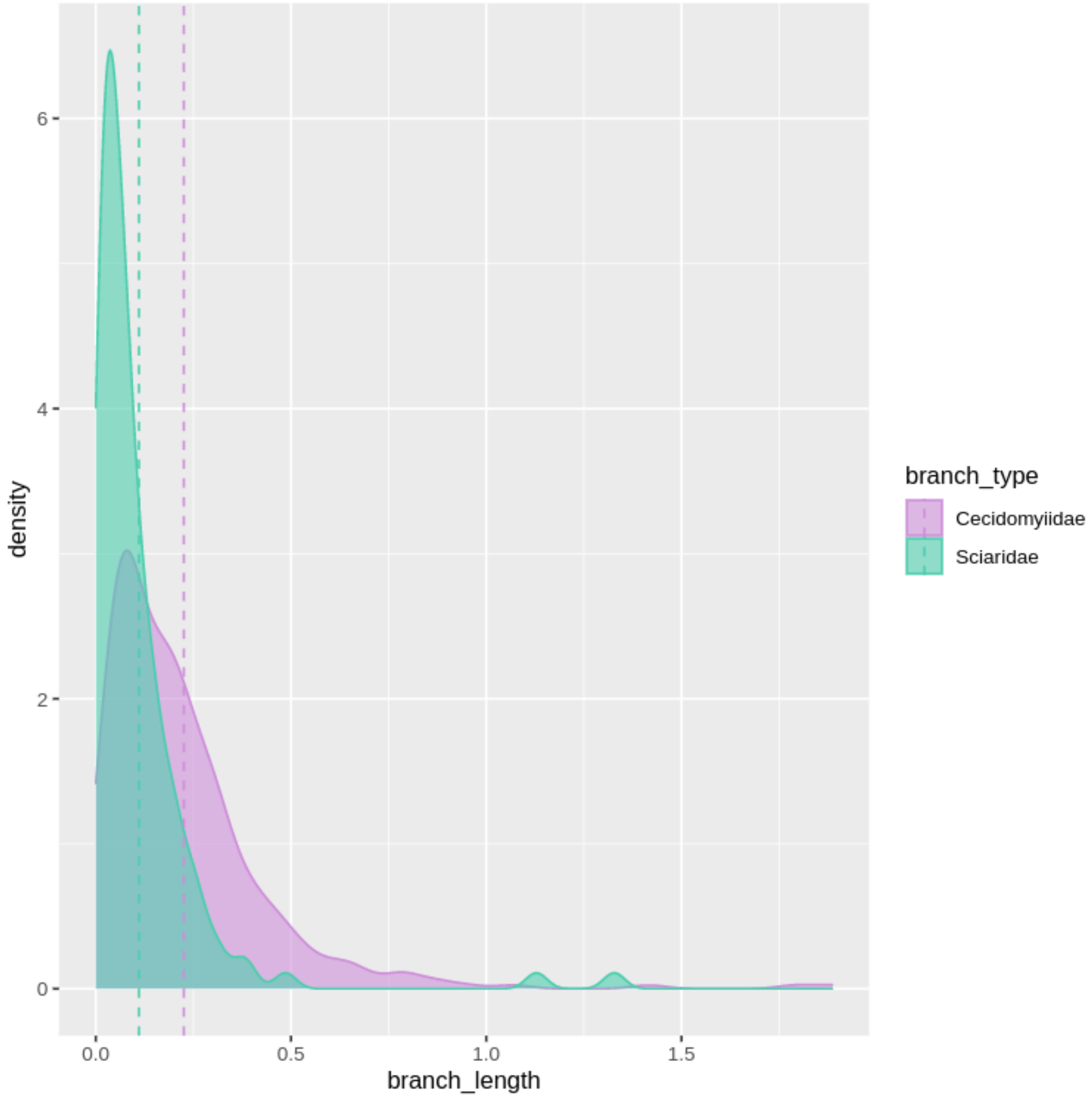

S10 Fig. Terminal branch length distribution of GRC genes; Branch length distribution of GRC copies of BUSCO genes plotted with respect to the phylogenetic position (at family level), means shown by dashed lines. Branch lengths of BUSCO genes on GRCs within Cecidomyiidae (violet) are significantly longer than branches found within Sciaridae (teal; p-value < 0.0001) suggesting the genes on GRCs found within Sciaridae might be due to gene

duplications and translocations within Sciaridae after the GRCs were acquired. Location of data used to generate figure is specified in **S1 Table.**
