# Supplementary material for: Gene-rich germline-restricted chromosomes in black-winged fungus gnats evolved through hybridization
Source: PLoS Biol. 2022 Feb 25;20(2):e3001559. doi: 10.1371/journal.pbio.3001559 (PMC8906591; doi:10.1371/journal.pbio.3001559)
Supplement: S11 Fig — (A) Scatterplot of the amino acid identity for GRC genes that had a reciprocal blast hit to both the B. coprophila core genome (x-axis) and the M. destructor core genome (y-axis). The majority of genes had a greater similarity to the M. destructor genome. (B) For genes that had a greater similarity to the B. coprophila genome (teal background in A), a histogram of the homolog identity to the B. coprophila core genome gene, with (C) showing a histogram of the homolog identity to the M. destructor core genome gene for genes that had a greater similarity to the M. destructor genome (purple background in A). (D) Histogram showing the number of reciprocal blast hits for GRC genes to the B. coprophila core genome, the number of unique hits (i.e., taking only one hit for each GRC gene with the highest identity), the number of reciprocal blast hits for GRC genes to the M. destructor core genome, the number of unique hits, and the number of GRC genes which had a reciprocal blast hit in both the M. destructor and B. coprophila genome. The genes in the last category were used for plots A,B, and C. Note that comparisons of reciprocal blast hits between these 2 genomes should be taken with a grain of salt, as the M. destructor genome was not annotated in the exact same way as we annotated the B. coprophila genome. However, we would not expect this to substantially affect the patterns of homology shown above. Location of data used to generate this figure is specified in S1 Table. GRC, germline-restricted chromosome. (PDF) [file pbio.3001559.s020.pdf]

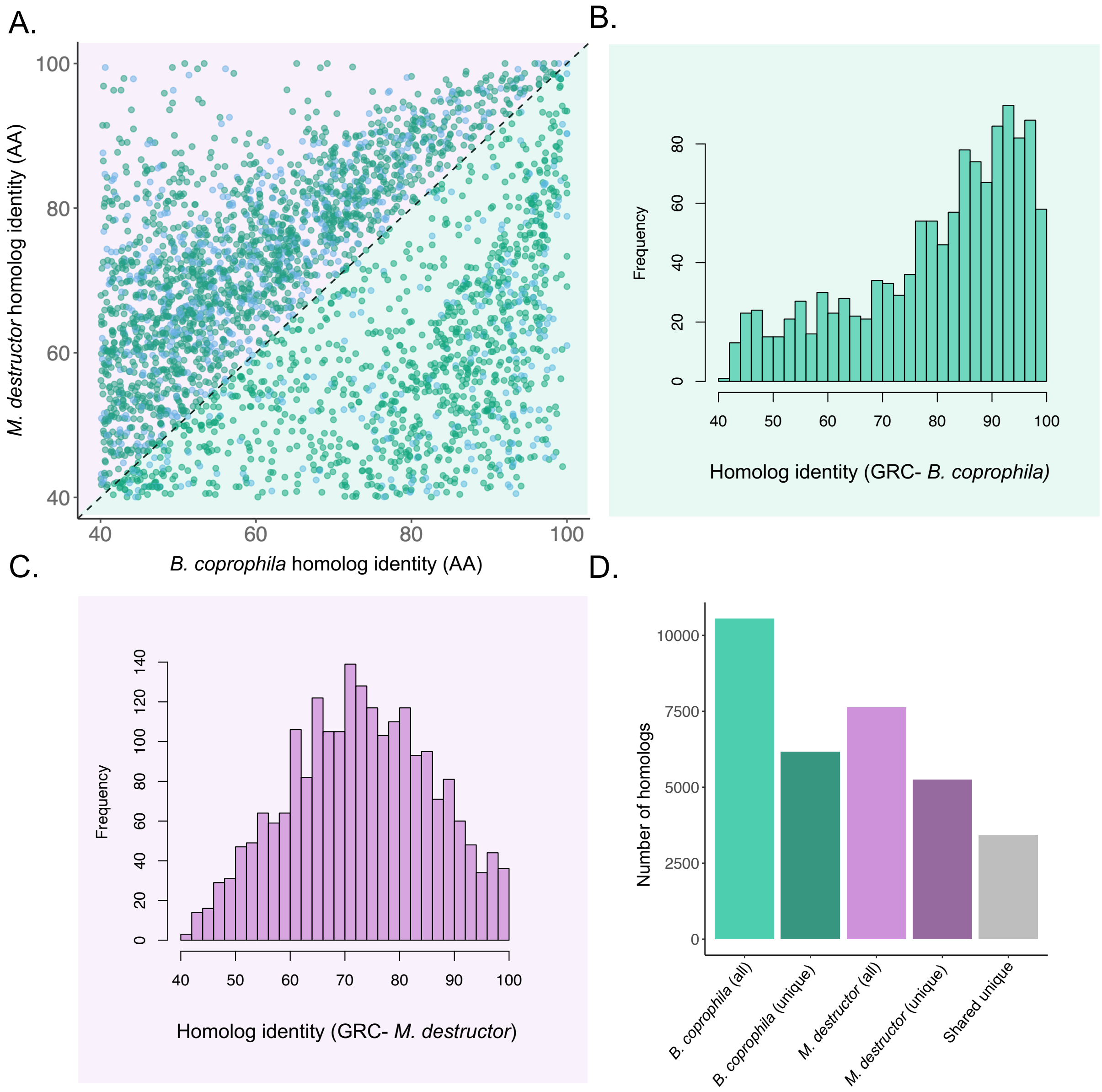

S11 Fig. Comparison of GRC homolog amino acid identity to *M. destructor* or *B. coprophila* core genes. A. Scatterplot of the amino acid identity for GRC genes that had a reciprocal blast hit to both the *B. coprophila* core genome (x-axis) and the *M. destructor* core genome (y-axis). The majority of genes had a greater similarity to the *M. destructor genome.* **B.** For genes that had a greater similarity to the *B. coprophila* genome (teal background in A), a histogram of the homolog identity to the *B. coprophila* core genome gene, with **C.** showing a histogram of the homolog identity to the *M. destructor* core genome gene for genes that had a greater similarity to the *M. destructor* genome (purple background in **A**). **D.** Histogram

showing the number of reciprocal blast hits for GRC genes to the *B. coprophila* core genome, the number of unique hits (i.e. taking only one hit for each GRC gene with the highest identity), the number of reciprocal blast hits for GRC genes to the *M. destructor* core genome, the number of unique hits, and the number of GRC genes which had a reciprocal blast hit in both the *M. destructor* and *B. coprophila* genome. The genes in the last category were used for plots A,B, and C. Note that comparisons of reciprocal blast hits between these two genomes should be taken with a grain of salt, as the *M. destructor* genome was not annotated in the exact same way as we annotated the *B. coprophila* genome. However, we would not expect this to substantially affect the patterns of homology shown above. Location of data used to generate this figure is specified in **S1 Table.**
